# Differential alternative splicing coupled to nonsense-mediated decay of mRNA ensures dietary restriction-induced longevity

**DOI:** 10.1038/s41467-017-00370-5

**Published:** 2017-08-21

**Authors:** Syed Shamsh Tabrez, Ravi Datta Sharma, Vaibhav Jain, Atif Ahmed Siddiqui, Arnab Mukhopadhyay

**Affiliations:** 1 Molecular Aging Laboratory, National Institute of Immunology, Aruna Asaf Ali Marg, New Delhi, 10067 India; 2 Amity Institute of Biotechnology (AIB), Amity University Haryana, Panchgaon, Manesar, Haryana 122413 India

## Abstract

Alternative splicing (AS) coupled to nonsense-mediated decay (AS-NMD) is a conserved mechanism for post-transcriptional gene regulation. Here we show that, during dietary restriction (DR), AS is enhanced in *Caenorhabditis elegans* and mice. A splicing mediator *hrpu-1* regulates a significant part of these AS events in *C. elegans*; knocking it down suppresses DR-mediated longevity. Concurrently, due to increased AS, NMD pathway genes are upregulated and knocking down UPF1 homologue *smg-2* suppresses DR lifespan. Knockdown of NMD during DR significantly increases the inclusion of PTC-containing introns and the lengths of the 3′UTRs. Finally, we demonstrate that PHA-4/FOXA transcriptionally regulates the AS-NMD genes. Our study suggests that DR uses AS to amplify the proteome, supporting physiological remodelling required for enhanced longevity. This increases the dependence on NMD, but also helps fine-tune the expression of metabolic and splicing mediators. AS-NMD may thus provide an energetically favourable level of dynamic gene expression control during dietary restriction.

## Abstract (web summary)

Alternative splicing coupled to nonsense-mediated decay (AS-NMD) is a conserved mechanism for post-transcriptional gene regulation. Here, the authors provide evidence that AS-NMD is enhanced during dietary restriction (DR) and is required for DR-mediated longevity assurance in *C. elegans*.

## Introduction

The alternative processing of precursor messenger RNA (mRNA) to generate multiple isoforms is a well-conserved mechanism to increase complexity of the transcriptome and, consequently, the diversity of proteins in multicellular organisms, thereby bypassing the limitations posed by a finite genome^1–3^. A pre-mRNA that is made up of exons interspersed with introns is recognized by a large multiprotein splicing complex, which removes the intervening introns to produce the mature mRNA. During this splicing step, a pre-mRNA can produce one or more functional transcripts through a process called alternative splicing (AS). Most AS events are now classified into seven splicing patterns: cassette exons, alternative donors, alternative acceptors, alternative initiation, alternative termination, mutually exclusive, and retained introns^4^.

AS alters the repertoire of proteins under different physiological conditions, thereby helping an organism to adapt to the changing environment. For example, isoforms generated by AS may have different binding affinities to ligands or other interacting proteins^5^. In case of transcription factors, a change in binding domain can prevent or enhance the interaction of the protein with DNA, modifying its regulatory role during transcription^6^. AS may also lead to changes in localization of proteins^7^, their post-translational modifications^8, 9^ or alter their enzymatic activity^3^. As a result, even subtle changes in splicing patterns can lead to various debilitating diseases^2^. Accordingly, many age-associated diseases are characterized by changes in AS patterns^10–12^.

Human studies have shown that around 92–94% of the genes are alternatively spliced,^13^ and of these, 40% of AS events can generate in-frame premature termination codons (PTCs), which are natural targets of the nonsense-mediated decay (NMD) pathway, a post-transcriptional RNA surveillance mechanism^14, 15^. The degradation of PTC-containing transcripts is important to prevent the production of truncated proteins that can have a dominant-negative effect. NMD was thought to have evolved solely to degrade PTC-containing transcripts that arise either from mutations at the DNA level or from inefficient or inaccurate splicing^1^. However, now it is becoming evident that cells have also adapted this mechanism to control gene expression by coupling AS with NMD (AS-NMD), even after the mRNA has been transcribed^16^. AS-NMD thus plays an important role in regulating the function of a gene by increasing the expression of a non-functional NMD-targeted isoform of the active gene, thereby decreasing the translation of the protein. This coupling of AS and NMD to regulate protein expression is often called regulated unproductive splicing and translation (RUST)^15^. This mechanism is well-conserved across species and is predominantly used by various splicing factors to autoregulate their expression, including the serine and arginine-rich class of splicing factors^17–19^.

With the advent of next-generation sequencing (NGS) technologies, there has been a growing interest to delineate the role of RNA-regulatory mechanisms in aging and longevity assurance^20–26^. However, the role of AS-NMD in these processes is yet unknown. Dietary restriction (DR), also referred to as caloric restriction (CR), is the only known intervention that increases lifespan across species and delays onset of various age-associated pathologies^27, 28^. DR entails a large-scale reprogramming of the cellular metabolic processes that supports the increased lifespan and requires an extensive transcriptional and post-transcriptional response^20, 22, 29, 30^.

Here we show that, during DR in worms, AS is dramatically increased in genes that are involved in metabolism, RNA-regulatory processes, cellular signalling and protein processing. The exons that are preferentially used during DR are enriched for ATPase and kinase domains, enhancing the biological functions of the proteins. This post-transcriptional response is conserved in hippocampus of mice undergoing CR where genes involved in mRNA splicing and processing, chromatin modifications, signal transduction, metabolism and cellular transport are alternatively spliced. In worms, we identify a splicing mediator, *hrpu-1* (*y41e3.11*) that regulates a major portion of these AS events, making it an important modulator of DR lifespan. Concurrently, we show that NMD genes are upregulated and the process is required for DR-induced longevity assurance. The DR-specific pioneering transcription factor PHA-4/FOXA was found to transcriptionally regulate the AS-NMD genes, like *hrpu-1* and *smg-2*. Finally, we show that genes that incorporate PTC due to AS are enriched in biological processes of metabolism, mRNA splicing and innate immunity; these genes are actively regulated or degraded during DR. Together, our study identifies AS-NMD as a mechanism of gene regulation with a positive bearing on lifespan during DR.

## Results

### Elevated levels of AS during DR in ***Caenorhabditis elegans***

**Fig. 1:**
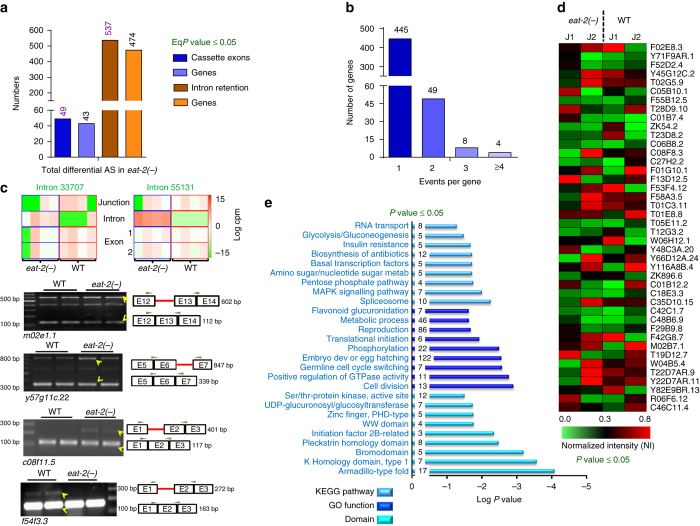
Increased alternative splicing during DR in *C. elegans*. **a** AS events in day 3 *eat-2(ad1116)* (shown as *eat-2(-)*) worms, as exhibited by the differential usage of cassette exons (CE; *dark blue bars*) or by intron retention (IR; *dark brown bars*). Total spliced-in and spliced-out events in *eat-2(ad1116)* are shown. The genes that these events correspond to are indicated in the *lighter shades*. Statistical test: Irwin–Hall *P* value summarization was used on each individual splicing event (details in the Methods section). Four biological replicates were used for analysis. **b** Distribution of the number of AS events per gene during DR. Both CE and IR events are considered together. **c** *Upper panels* showing representative images of AS involving intron retention. For this, two terms were considered, reads mapping to the introns and that of the skipping junction when the intron is not included, while ensuring that flanking exons are expressed. Each *blue* or *red box* represents four biological replicates of the RNA-seq. In case of Introns 33707 and 55131, the read counts mapping to the intron or the junction were reciprocal in *eat-2(ad1116)* vs. WT. *Lower panels*: The above intron retention events belonging to *m02e1.1* and *y57g11c.22* are validated by RT-PCR. Two other AS events belonging to *c08f11.5* and *f54f3.3* are also validated below. In case of *m02e1.1*, *y57g11c.22* and *c08f11.5*, the isoforms retaining the intron increase in *eat-2(ad1116)*, whereas the reverse happens in *f54f3.3*. The two lanes for each sample are biological replicates. *Yellow arrows* indicate two alternatively spliced isoforms that show differential expression in WT and *eat-2(ad1116)*. Similar results were observed in at least three biological replicates. *cpm* counts per million. **d** Heat map depicting differential AS of top 42 genes in *eat-2(ad1116)*. J1 and J2 indicate two junctions of the same gene showing highest significant differences in their splicing indices. Statistical analysis was performed using two-tailed unpaired *t-*test with Sidak–Bonferroni correction for individual event. Data from four biological replicates. **e** KEGG pathway, Gene Ontology (GO) term enrichment and protein domains were determined by DAVID Functional Annotation tool using differentially AS genes. The numbers within the *blue bars* represent genes detected in the category. The listed pathways have a *P* value ≤ 0.05, Fisher Exact Test

DR entails a massive reprogramming of organismal physiology to support enhanced longevity and health span. To study DR in *C. elegans*, genetic mutations in the acetylcholine receptor gene *eat-2* is often utilized^31^. Mutations in *eat-2* (as in *eat-2(ad1116)* or *eat-2(ad465)*) lead to decreased pharyngeal pumping. As a result, these worms consume less bacteria and display many DR-like attributes, including metabolic reprogramming, enhanced autophagy, increased beta oxidation and cellular detoxification, reduced fecundity and increased lifespan. To reveal the genome-scale reorganization of the transcriptome in the form of altered AS, analysis of RNA-sequencing (RNA-seq) data were performed to compare wild-type (WT) N2 Bristol and *eat-2(ad1116)* at day 1^22^ or day 3 of adulthood^20^. Of the seven AS patterns known, we focussed on two types of AS events, namely, cassette exons (where an exon is spliced-in or spliced-out) and intron retention (where an intron is retained in one isoform under a certain condition) when analysing the day 3 data (Fig. 1). We utilized several terms to pin-point each AS event. In the case of cassette exons, four different terms were considered together, i.e., reads mapping to the cassette exon and its flanking junctions as well as the skipping junction formed due to the exclusion of the cassette exon. We followed the logic that if an exon is included in a transcript, then the exon along with its flanking junctions should be expressed, without expression of the skipping junction and vice versa. In case of intron retention, we focussed on two terms, i.e., reads that mapped to the intron itself as well as to the skipping junction created when the intron is excised, while taking into account that the flanking exons are also expressed. The two-tailed *P* values obtained from limma for all the associated terms were converted to one-tailed *P* values and finally summarized using Irwin–Hall method to get a combined *P* value (Eq*P* values) (details provided in the Methods section). We found 45 significant events of cassette exons (in 41 genes) that were spliced-in, while 4 events (in 3 genes) were spliced-out in *eat-2(ad1116)*, totalling to 49 events of AS (Fig. 1a, Supplementary Data 1). Similarly, we observed 312 intron retention events (in 291 genes) that were significantly enhanced while 225 events diminished (occurring in 210 genes) in *eat-2(ad1116)*, totalling to 537 AS events (Fig. 1a, Supplementary Data 2). Most of the genes undergoing AS (cassette exon as well as intron retention) had only one AS event (Fig. 1b). Since DR has been reported to upregulate gene transcription^22, 29^, we ensured that the observed AS events are not simply due to the changes in gene expression (Supplementary Fig. 1A). When we plotted the relative reads per kilobase of exon per million reads mapped (RPKM) of an AS event (*eat-2(ad1116)* vs. WT) or that of the corresponding gene in which the event takes place, we observed that they do not overlap in most cases (Supplementary Fig. 1A). Some representative events of exon and intron retention along with PCR validation are shown in Fig. 1c. To further visualize the AS events, we calculated the splicing index (SI) of each junction (as explained in Methods section) in WT and *eat-2(ad1116)*. We found that 578 junctions (contributed by 534 genes) have a SI of >1.0 (minimum read counts 4; *P* value ≤ 0.05 by two-tailed unpaired *t-*test with Sidak–Bonferroni correction) in case of day 3 DR samples while the numbers were 547 (contributed by 494 genes) on day 1 samples (Fig. 1d, Supplementary Fig. 1B, Supplementary Data 3, 4), indicating that the junction usage in WT and *eat-2(ad1116)* for these genes were altered significantly. Some of the events on day 1 were validated in Supplementary Fig. 1C. Together, this advocates for the fact that differential AS may be playing a vital role in regulating the expression of many genes during DR.

Large-scale AS may be required to support the metabolic and physiological reprogramming that ensures increased longevity during DR. This assumption is supported by the observation that genes undergoing AS during DR are enriched for important biological pathways pertaining to metabolism, RNA transport and processing, splicing, translation, etc. (Fig. 1e, Supplementary Data 5). These genes function in reproduction, larval development, cell division, gonad and germ cell development as well as in different metabolic processes (Fig. 1e, Supplementary Data 5). Further, in order to understand the functional significance of the preferentially used exons as a result of AS, we determined the domains encoded by them (as explained in Methods section). We found that different ATPase and kinase domains are enriched in these exons compared to randomly selected ones, signifying that these new isoforms may gain important biological capabilities to support DR-mediated reprogramming (Supplementary Fig. 1D, Supplementary Data 6). Altogether, our data show that a large number of genes are alternatively spliced to generate different isoforms that may be functionally contributing to longevity assurance under DR.

### Differential AS is enhanced in mice undergoing DR

**Fig. 2:**
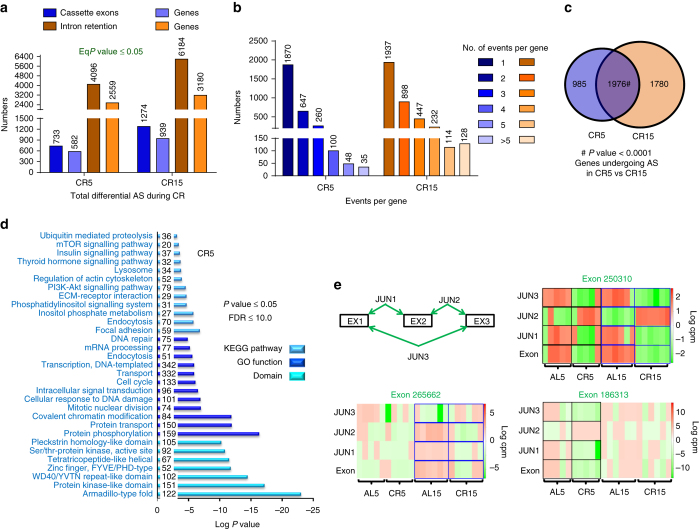
Alternative splicing is increased in the hypothalamus of mice undergoing CR. **a** Alternative splicing events in mice on caloric restriction (CR) for 5 or 15 months (CR5 or CR15), as exhibited by the differential usage of cassette exons (CE; *dark blue bars*) or by intron retention (IR; *dark brown bars*). Spliced-in and spliced-out events are considered together to represent the total number of differential AS events (CE and IR only) during CR. The genes that these events correspond to are indicated in the *lighter shades*. Statistical test: Irwin–Hall *P* value summarization was used on each individual splicing event (details in the Methods section). Data are from five (for CR5 or AL5) or six (for CR15 or AL15) biological replicates. **b** Distribution of the number of AS events (both CE and IR considered together) per gene in CR5 and CR15. **c** Significant overlap between genes undergoing AS during early (CR5) and late (CR15) phases of CR. *P* value determined by hypergeometric test. **d** KEGG pathway, Gene Ontology (GO) term enrichment and protein domains were determined by DAVID Functional Annotation tool using differentially AS genes in mice under CR diet for 5 months. The listed pathways have a *P* value ≤ 0.05 (Fisher Exact Test) and FDR<10.0. The numbers within the *blue bars* represent genes detected in the category. **e** Representative images of AS involving cassette exons. For this, four terms were considered, reads mapping to the cassette exon (Exon), the flanking junctions (JUN1, JUN2) and that of the skipping junction when the exon is not included (JUN3). Each box represents five (for CR5 or AL5) or six (for CR15 or AL15) biological replicates in the RNA-seq experiments. In case of Exon 250310, the read counts mapping to the exon or the JUN3 were reciprocal in AL5 vs. CR5 as well as in AL15 vs. CR 15. On the other hand, the reciprocal relationship was observed either in case of early (Exon 186313) or late (Exon 265662) CR. *cpm* counts per million

Next, we asked whether the increased AS observed in *C. elegans* is conserved in mammals. We carried out metadata analysis of an RNA-seq experiment performed using the hippocampus of mice maintained on AL (ad libitum) or CR diet for 5 or 15 months (AL5, CR5, AL15 or CR15)^32^. Interestingly, we find significant differential AS in the hippocampus of CR mice as compared to AL in utilizing cassette exon or intron retention (Fig. 2a, e, Supplementary Fig. 2C, Supplementary Data 7–10). On comparing CR5 with AL5 (represented as CR5 in the Fig. 2), we identified 733 cassette exons (occurring in 582 genes) generated by differential AS (Fig. 2a, Supplementary Data 7). Similarly, we found 4,096 intron retention events in 2,559 genes during this early stage of CR (Fig. 2a, Supplementary Data 8). Some representative AS events are shown in Fig. 2e and Supplementary Fig. 2C. Distribution of AS events per gene (Fig. 2b) shows that, although most genes have a single AS event, multiple AS events per gene were also not uncommon. We observed a further increase in AS events on long-term CR (CR15), both in case of cassette exons as well as intron retentions (Fig. 2a, Supplementary Data 9, 10). Additionally, we found a significant overlap between AS events (Supplementary Fig. 2A) or their corresponding genes (Fig. 2c) that undergo differential AS during early (CR5) and later (CR15) stages of CR. These point to the shared as well as distinct post-transcriptional response to short- and long-term DR. Like in the case of worm DR, we ensured that the observed AS events are not simply due to the changes in gene expression (Supplementary Fig. 2B).

Further, when we analysed the genes that were undergoing AS during early CR, we found them to be enriched for functions related to metabolism, cellular signalling, mRNA processing, protein processing and transport as well as endocytosis, similar to *C. elegans* (Fig. 2d, Supplementary Data 5). Also, genes involved in similar pathways undergo AS in later stages of CR (Supplementary Fig. 2D, Supplementary Data 5). This was also apparent when we performed Gene Ontology (GO) analysis (Supplementary Fig. 2E, Supplementary Data 5) of the 1,976 AS genes that overlap in CR5 and CR15 (Fig. 2c). Together, AS is increased during DR in both *C. elegans* and mice, establishing it to be a conserved response to nutrient restriction.

### *Hrpu-1*, a splicing mediator required for DR lifespan

**Fig. 3:**
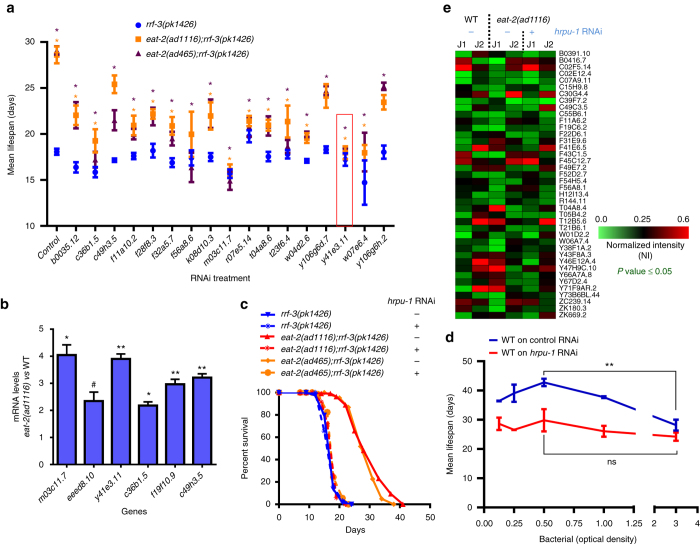
Splicing mediators are required for DR-mediated lifespan extension. **a** Specific requirement of splicing mediators in DR-mediated lifespan extension. Mean lifespans of *eat-2(ad1116);rrf-3(pk1426)* or *eat-2(ad465);rrf-3(pk1426)* when splicing factor genes were knocked down by RNAi. The *rrf-3(pk1426)* background makes *eat-2* mutants hypersensitive to RNAi. Averages of two or more biological replicates are shown. *P* values determined by unpaired two-tailed *t-*test between the control and gene-of-interest RNAi for each individual strain. *Asterisk* (*) indicates significant differences with *P* ≤ 0.05; *orange asterisk* is for *eat-2(ad1116);rrf-3(pk1426)*, *purple asterisk* is for *eat-2(ad465);rrf-3(pk1426)*; lifespan suppression in *rrf-3(pk1426)* was not significant in all cases. *Asterisk* in case of control RNAi shows that the lifespan of *eat-2(ad1116);rrf-3(pk1426)* or *eat-2(ad465);rrf-3(pk1426)* is significantly different from *rrf-3(pk1426)*. Lifespan data provided in Supplementary Table 2. Experiments were repeated at least three times. *Red box* indicates *y41e3.11* gene that we followed further. **b** qRT-PCR to detect upregulation of splicing mediator genes in *eat-2(ad1116)*. Expression levels were normalized to *actin* and compared to WT. Average of two biological replicates. *Asterisks* indicate statistically significant differences to the corresponding WT samples as calculated by unpaired two-tailed *t*-test (*P ≤ 0.05; **P < 0.01, ^#^ *P* = 0.0556). **c** Representative lifespans (one of three or more) of *eat-2(ad1116)* and *eat-2(ad465)* when grown on *y41e3.11* (*hrpu-1*) RNAi (indicated by +) from L4 onwards. Control (vector) RNAi indicated by negative (−). Lifespan data are provided in Supplementary Table 2. **d** A bDR assay using WT grown on control or *hrpu-1* RNAi. Control RNAi-grown worms produced a *bell-shaped graph*, while the ones grown on *hrpu-1* failed to do so. An average of two biological replicates is shown. Bacterial culture of OD_600_ = 3.0 was the starting concentration for serial dilution. Additionally, the increase in lifespan when WT on control RNAi were maintained at OD_600_ = 0.5 was significant as compared to OD_600_ = 3.0; the difference in case of WT on *hrpu-1* RNAi was not. Unpaired two-tailed *t-*test, **P ≤ 0.01, *ns* non-significant. The bDR lifespan data are provided in Supplemental Table 3. **e** Reversal of differential alternative splicing pattern of *eat-2(ad1116)* upon knockdown of *hrpu-1* by RNAi. J1 and J2 indicate two junctions of the same gene showing the highest significant differences in their splicing indices. Four biological replicates were used for RNA-seq. Statistical analysis was performed on NI using two-tailed unpaired *t-*test with Sidak–Bonferroni correction for individual event. (*Error bars* = SEM)

On finding a large increase in splicing and AS events in *eat-2(ad1116)*, we asked whether splicing mediators are important for the enhanced lifespan. To this end, we performed a targeted RNAi screen using 83 splicing mediators in *C. elegans*, collated on the basis of homology search and published literature. On systemically knocking them down using RNAi in WT or *eat-2(ad1116)*, we found 17 RNAi that significantly suppressed the enhanced lifespan of *eat-2(ad11116)* with marginal effect on WT (Fig. 3a, Supplementary Table 1, 2). A similar suppression was observed in case of *eat-2(ad465)* allele (Fig. 3a, Supplementary Table 2). Interestingly, in our RNA-seq data, the expression of several of these splicing factors were found to be upregulated in *eat-2(ad1116)* compared to WT (Supplementary Fig. 3A); we validated six of these using quantitative reverse transcriptase PCR (qRT-PCR) (Fig. 3b). We observed maximum lifespan suppression in case of the splicing factor *hrpu-1* (*y41e3.11*), although RNAi of *hrpu-1* had little effect on WT lifespan; so we followed this gene further. We verified that the expression of *hrpu-1* was downregulated significantly in both WT and *eat-2(ad1116)* on RNAi (Supplementary Fig. 3B). Interestingly, *hrpu-1* knockdown initiated at young adult stage was sufficient to suppress the DR lifespan completely (Fig. 3c); knocking it down during development shows some embryonic lethality (http://www.wormbase.org/species/c_elegans/gene/WBGene00012769). Importantly, *hrpu-1* was also required for another paradigm of DR, namely, bacterial dilution (bDR)^30, 33, 34^. In a bDR assay, populations of worms are fed with a decreasing quantity of bacteria. When the average lifespan of WT worms on each dilution is plotted, a bell-shaped curve is generated with one dilution showing maximum lifespan increase (Fig. 3d, Supplementary Table 3). However, when an important mediator of DR, such as *hrpu-1* is knocked down, a relatively flat graph is obtained; none of the concentrations is able to produce lifespan increase (Fig. 3d, Supplementary Table 3). Together, these experiments show that splicing mediators are upregulated during DR, possibly to support the increased need for AS and thus are required for the extended lifespan of DR worms.

### *Hrpu-1* regulates a part of the AS events in *eat-2(ad1116)*

We have shown that AS is upregulated during DR and knocking down *hrpu-1* results in complete suppression of *eat-2(ad1116)* and bDR lifespan. So we asked whether this splicing mediator is required for the increased AS observed in the strain. We grew *eat-2(ad1116)* on control or *hrpu-1* RNAi for 48 h post-L4 and performed RNA-seq analysis. We computed the differential AS index between the strains on control or *hrpu-1* RNAi and found that several of the AS events in *eat-2(ad1116)*, which were significantly different from WT, stood reverted (Fig. 3e, Supplementary Fig. 3D, Supplementary Data 11). Of the 638 genes with enhanced AS in *eat-2(ad1116)* compared to WT, 147 genes were regulated by *hrpu-1* (Supplementary Fig. 3C). These include genes coding for NMD (*smg-1*), chromatin modifiers (*nurf-1*, *set-2*), RNA processing proteins (*tsen-54*, *cpsf-1*, *pqe-1*), transcription factors (*nhr-57*, *nhr-61*, *ceh-88*, *nfi-1*, *egl-13*, *jun-1*, *klf-3*, *lin-39*, *tbx-33* and *tbx-43*), metabolic regulators (*daf-15*, Rictor orthologue; *sur-5*, acetoacetyl-CoA synthetase; *adr-1*, adenosine deaminase acting on RNA; *mans-3*, alpha-1,2-mannosidase; *moc-1*, molybdenum cofactor biosynthesis; *nceh-1*, neutral cholesterol ester hydrolase; *scrm-4*; phospholipid scramblase), autophagy and endocytosis mediators (*atg-2*, *atg-4.2*, *rme-8* and *vps-52*), transporters (*ifo-1*, *klc-2*, *klp-4*, *pept-2*, *amt-1*, *ncx-7*, *spe-41*), signal transducers (kinases like *unc-43*, *kin-14*; receptors like *ckr-2*, *gcy-28*, *srsx-34*, *ida-1*, *fshr-1*), DNA damage response proteins (*him-6/BLM*, *atl-1*, *pms-2*, *rad-54/RAD54L*, *msh-5*) and other unannotated genes. This data also partially explains the requirement of *hrpu-1* in *eat-2(ad1116)* as well as bDR lifespans and reaffirms the importance of AS during DR.

### NMD is required for DR-induced longevity

Upregulation of AS and, particularly intron retention, may result in aberrant inclusion of PTC-containing exons in the transcript, either due to mis-splicing or as a result of RUST^15, 35–37^. These inappropriate transcripts are identified and removed by the NMD pathway. The NMD pathway involves multiple proteins that are conserved and well characterized in model organisms. The up-frameshift proteins, UPF1, UPF2 and UPF3, comprises the core complex of NMD and were first discovered in yeast^38^. UPF1 is a member of the group I helicase family and has an RNA-binding property, while UPF2 and UPF3 are part of the exon junction complex. The other components of the NMD pathway, SMG-1, SMG-5, SMG-6 and SMG-7, were later discovered in *C. elegans* through a genetic screen^39^. Sequence alignments revealed that SMG-2 is homologous to UPF1, SMG-3 to UPF2 and SMG-4 to UPF3. UPF1 (*smg-2* in worms) is the central regulator of this pathway and inactivating it increases the concentration of PTC-containing transcripts without affecting the levels of WT isoforms^40^.

**Fig. 4:**
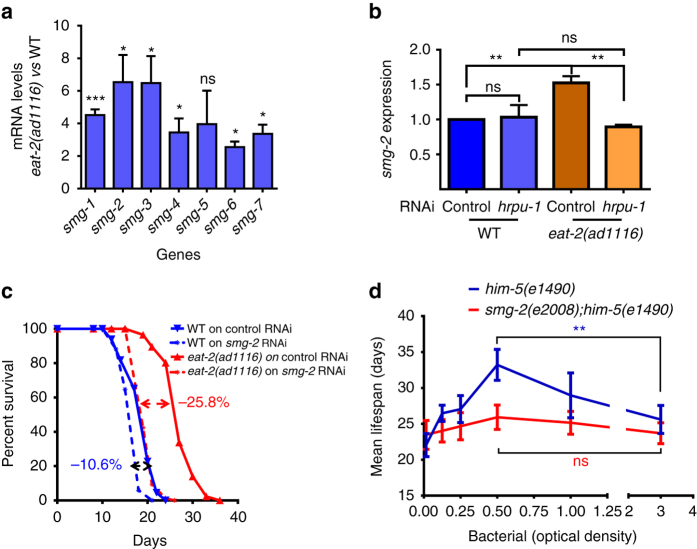
MRNA surveillance pathway is upregulated and required for DR-mediated longevity. **a** qRT-PCR showing that genes of NMD pathway are upregulated in *eat-2(ad1116)* at day 1 of adulthood. Expression levels were normalized to *actin* and compared to WT worms. Averages of three biological replicates are shown (*Error bars* = SEM). *Asterisks* indicate statistically significant differences to the corresponding WT samples, as calculated by unpaired two-tailed *t-*test (*P ≤ 0.05; **P ≤ 0.01, ***P ≤ 0.001, *ns* non-significant). **b** The expression of *smg-2* is upregulated in *eat-2(ad1116)* compared to WT, in a *hrpu-1*-dependent manner. The samples were collected on day 3 of adulthood. Data shown are average of three biological replicates. (*Error bars* = SEM). Statistical analysis was performed using unpaired two-tailed *t-*test. **P ≤ 0.01, *ns* non-significant. **c** Lifespan of WT and *eat-2(ad1116*) grown on control or *smg-2* RNAi. One out of the three biological replicates are shown. Percentage of suppression of lifespan is indicated for each strain. Lifespan data for each individual experiment are provided in Supplementary Table 4. **d** *Bell-shaped curve* generated by plotting average lifespans of *him-5(e1490)* grown in the presence of different dilutions of bacteria. The *smg-2(e2008);him-5(e1490)* mutant worms failed to produce similar curve. An average of three independent biological replicates is shown. *Error bars* are SEM. Two-way ANOVA revealed that the lifespan extension of *him-5(e1490)* worms across the bacterial dilution range was significantly different from that of *smg-2(e2008);him-5(e1490)* (*P* ≤ 0.05). Bacterial culture of OD_600_ = 3.0 was the starting concentration for serial dilution. Additionally, the increase in lifespan when *him-5(-)* was maintained at OD_600_ = 0.5 was significant as compared to OD_600_ = 3.0; the difference in case of *smg-2(-);him-5(-)* was not. **P ≤ 0.01, *ns* non-significant; unpaired two-tailed *t-*test. All the mean lifespan and statistical analysis are provided in Supplementary Table 4

Importantly, in our RNA-seq experiments, the transcript levels of most of the NMD components (*smg-1–4*, *smg-6* and *smg-7*) were also found to be significantly upregulated in *eat-2(ad1116)* that we validated using qRT-PCR in *eat-2(ad1116)* as well as *eat-2(ad465)* (Fig. 4a, Supplementary Fig. 4). More importantly, we found that the increased expression of *smg-2* in *eat-2(ad1116)* was dependent on *hrpu-1* (Fig. 4b), providing evidence that AS and NMD are coupled downstream of DR.

Next, we asked whether the lifespan of *eat-2(ad1116)* requires functional NMD. For this, we knocked down *smg-2* in WT or *eat-2(ad1116)* and observed significantly greater suppression in the latter (Fig. 4c, Supplementary Table 4). Next, we tested the requirement of *smg-2* in bDR, the non-genetic mode of DR^33, 34^. The worms having a WT copy of *smg-2* produced the typical bell-shaped curve when the average lifespan was plotted against the bacterial concentrations (optical density at 600 nm (OD_600_)), showing that the bDR regime indeed worked (Fig. 4d, Supplementary Table 4). However, the *smg-2(e2008)* mutants failed to exhibit a graded response to the dilution of bacterial feed and generated a flattened survival curve (Fig. 4d, Supplementary Table 4). These experiments show that NMD is required for multiple DR paradigms to positively influence lifespan.

### Transcripts targeted by AS-NMD during DR

**Fig. 5:**
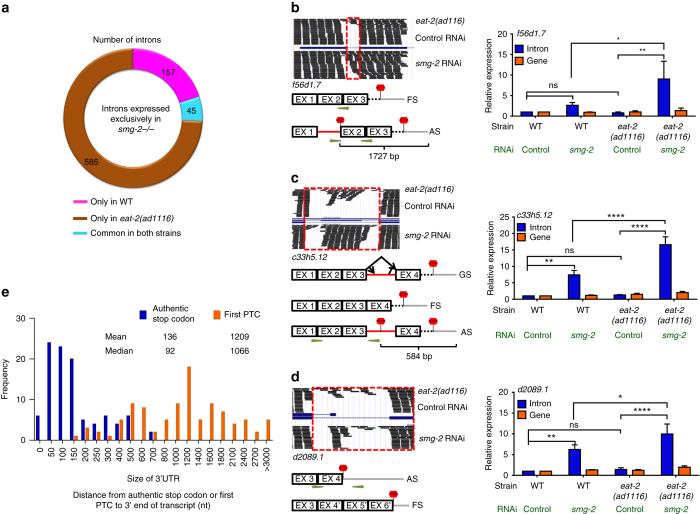
Genes targeted by AS-NMD during DR. **a** Number of introns retained when WT or *eat-2(ad1116)* were grown on *smg-2* RNAi. **b**–**d** UCSC Browser views show parts of the *f56d1.7*, *c33h5.12* or *d2089.1* genes and mapped sequencing reads when *eat-2(ad1116)* worms were grown on control or *smg-2* RNAi (*upper panel*). A graphical representation (GR) of the two alternate forms (*FS*- functionally spliced with normal stop codon; *AS*-alternatively spliced with a PTC) of the transcripts is shown below the browser view. The qRT-PCR validation of the events, along with the expression of the genes, is presented on the *right-hand side*. In **b**–**d**, differential intron retention (*blue bars*) was detected in *eat-2(ad1116)* and WT, grown on *smg-2* or control RNAi. Expression levels were normalized to *actin* and compared to WT on control RNAi. Averages of three biological replicates are shown (*error bars* = SEM). *Asterisks* indicate statistically significant differences as calculated using unpaired two-tailed *t-*test (*P ≤ 0.05; **P ≤ 0.01, ***P ≤ 0.001 and ****P ≤ 0.0001; *ns* non-significant). The changes in the expression of the respective genes (*orange bars*) were ns. **b** AS product with PTC is marked in the GR. Primer pairs were designed to quantify the expression of the intron-containing transcript. Distance between PTC and the start of poly-A site is indicated. **c** The gene structure (GS) of *c33h5.12* is presented below the browser view. Rest as (**b**). **d** The *d2089.1* gene has AS of the introns in its 3′UTR to generate transcripts with different UTR lengths. The FS in this case has exons and introns that are utilized as 3′UTR in the AS transcript; this results in an extended 3′UTR. Primer pairs were designed to quantify the expression of the 3′UTR of the AS transcript; they do not amplify the FS transcript. **e** Frequency distribution of the distance (nt) between the first PTC to the 3′ end of the transcript (*orange*) compared to the distance of normal stop codon to the 3′ end of the transcript of the same gene (*blue*). The graph shows 100 out of the 630 genes that retained an intron when *smg-2* was knocked down in *eat-2(ad1116)*

The alternatively spliced mRNAs that incorporate PTC are rapidly degraded by the NMD, although low levels may be detected even in *smg* ^+/+^ background^3^. However, these PTC-containing mRNAs become stabilized if the NMD is compromised. So we knocked down *smg-2* using RNAi in WT or *eat-2(ad1116)* and performed RNA-seq analysis focussing on one form of the AS events, i.e., intron retention. In these cases, AS leads to the mis-incorporation of an intron (complete or partial sequence) between a pair of exons or in the 3′ untranslated region (UTR). Interestingly, we found that a large number of introns were retained exclusively on knocking down *smg-2* in *eat-2(ad1116)* (585 introns, contributed by 555 genes), compared to WT (156, contributed by 152 genes) (Fig. 5a, Supplementary Data 12). We also found that, even in the presence of functional NMD, introns were retained; but on *smg-2* knockdown, their expression significantly increased in *eat-2(ad1116)* (Supplementary Data 12). We validated the retention of introns using qRT-PCR for several genes (Fig. 5b–d, Supplementary Fig. 5A–C) that showed intron retention in our RNA-seq analysis. We also ensured that our observation of increased intron retention was not simply due to an overall increase in the expression of the gene. As seen in Fig. 5b–d and Supplementary Fig. 5A–C, the expression of these genes does not change significantly. This approach showed that indeed an increase in the expression of an alternate intron-containing isoform had taken place when *smg-2* was knocked down.

We witnessed different forms of intron retention during DR. For example, for *f56d1.7* and *c50f4.1*, a single intron is retained in each case that produced a PTC (Fig. 5b, Supplementary Fig. 5A). On the other hand, in *c33h5.12*, three isoforms may be formed due to AS (Fig. 5c) by retaining a part or the whole of a large intron. For this gene, we found that an alternate splice start or stop site is stabilized on knocking down *smg-2*, leading to the retention of a PTC-containing intron. In an interesting example of intron retention in the 3′UTR, AS leads to the incorporation of PTC and significantly lengthens the UTR of the splicing factor *d2089.1* (*rsp-7*, Fig. 5d). In all the above cases, the length of 3′UTR is significantly extended and such transcripts are known to be NMD targets^41, 42^. We found that, in *C. elegans*, the median size of 3′UTR is 120 bp (Supplementary Fig. 5D). But when we performed in silico translation of 100 mRNAs that retained a complete intron during DR, we found that all of them had PTCs that resulted in extension of their 3′UTR from a median value of 92 to that of 1,066 bp (Fig. 5e). Thus these transcripts are actively degraded by NMD and could only be readily detected in *smg* ^*−/−*^ background.

Finally, we wanted to understand the biological processes being regulated by AS-NMD during DR. For this, we determined the GO terms and KEGG pathways as well as domains of the genes that are targeted by AS-NMD. We found that functions pertaining to RNA processing, metabolism and innate immunity are enriched (Supplementary Fig. 5E, Supplementary Data 5). Together, our data reveal that regulated use of AS-NMD during DR is important for controlling various biologically important processes to modulate lifespan.

### Transcription factor PHA-4/FOXA regulates AS-NMD genes

**Fig. 6:**
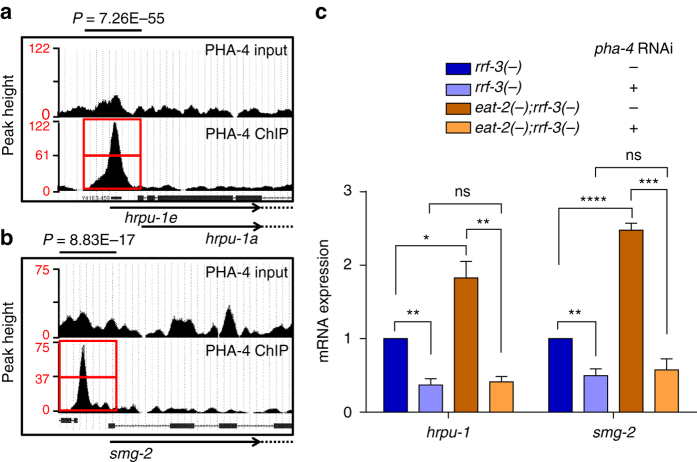
The DR-specific FOXA transcription factor PHA-4 regulates AS-NMD genes. **a**, **b** UCSC browser view of PHA-4/FOXA peaks on *hrpu-1* and *smg-2* promoters as determined by ChIP-seq analysis of *unc-119(ed3)III; wgIs37*(*OP37*) strain; data mined from MODENCODE and reanalysed using our bioinformatic pipeline. *Red boxes* indicate the promoter regions of *hrpu-1* and *smg-2* where peaks are observed. *Upper panel* shows peaks in input samples. Enriched peaks were identified using the peak calling algorithm MACS (v1.4.2). **c** QRT-PCR showing that *hrpu-1* and *smg-2* are upregulated in *eat-2(ad465)* in a *pha-4-*dependent manner. Expression levels were normalized to *actin*. Averages of three biological replicates are shown (*Error bars* = SEM). *Asterisks* indicate statistically significant differences, as calculated by unpaired two-tailed *t-*test (*P ≤ 0.05; **P ≤ 0.01, ***P ≤ 0.001, ****P ≤ 0.0001, *ns* non-significant). The *+ sign* indicates the presence of *pha-4* RNAi while *− sign* indicates control (vector) RNAi

DR requires a massive reprogramming of metabolism that translates into prolonged lifespan. Consequently, the Forkhead family transcription factors play important roles in DR-mediated longevity. However, the requirement for these transcription factors vary with the DR regimes being used. For example, *eat-2* mutant, *drl-1* knockdown and bacterial dilution in liquid media (bDR) require the FOXA transcription factor PHA-4^30, 33, 34, 43^. On the other hand, bacterial dilution on solid media requires the FOXO transcription factor DAF-16^44^. DAF-16 is not required for the DR regimes that we have used in this study. Since PHA-4 plays such an important role in controlling gene expression during DR^22, 30, 33, 43^, we asked whether PHA-4 controls the expression of *hrpu-1* and *smg-2* as well as other AS-NMD genes. For this, first we mined the MODENCODE data of PHA-4 ChIP-seq and found that PHA-4 binds to the promoter proximal regions of *hrpu-1* and *smg-2* as well as of *smg-1*, *smg-5*, *smg-6*, *c36b1.5* and *eeed8.10* (Fig. 6a, b, Supplementary Fig. 6A–C, E, F). Next, we knocked down *pha-4* using RNAi in WT and *eat-2(ad465)*. We found that both the basal and activated expression of *hrpu-1* and *smg-2* as well as the other AS-NMD genes are dependent on *pha-4* (Fig. 6c, Supplementary Fig. 6D, G). Together, PHA-4/FOXA transcriptionally regulates the expression of AS and NMD genes downstream of DR, acting as a major regulatory molecule.

## Discussion

DR benefits by delaying age-related diseases and increasing lifespan^28, 45–47^. In order to ensure these benefits, DR requires a massive cellular reprogramming in terms of coordinated transcriptional and post-transcriptional responses. New proteins need to be synthesized or new functional domains added to existing proteins to adapt to the changing environment while eliminating ones that are not required. Additionally, in this challenging milieu of limited nutrient availability, tight regulation of gene expression is of fundamental importance^22, 43^. AS-NMD is a conserved mechanism that alters the repertoire of proteins under different physiological conditions and helps in fine-tuning gene expression^1–3, 16^. In this study, we show that AS-NMD is required for DR-mediated lifespan extension.

AS is a major post-transcriptional regulatory mechanism that not only helps in amplifying the proteome by incorporating or excluding exons but also controls transcript abundance by coupling to NMD. In both worm and mice undergoing DR, we noticed multiple events of intron retention that may be a key source of PTC incorporation. Other AS events that we did not measure in this study, like alternative donors, alternative acceptors and alternative initiation, alternative termination^4^ may also potentially generate PTC-containing alternate transcript during DR. These events will activate the NMD pathway, as we have reported, to degrade the transcripts that may produce toxic truncated proteins. So if AS and NMD are indeed connected during DR, as reported in other conditions or systems^16, 48^, preventing AS during DR should also downregulate the transcription of NMD genes. Indeed, we show that *hrpu-1* RNAi in *eat-2(ad1116)* downregulates the transcription of *smg-2*, the UPF1 orthologue.

Protein translation is a highly energy-dependent process. During DR, the cellular system rationalizes energy use to cope with nutrient depletion. One of the way to do so is by reducing translation^49^ through lowered target of rapamycin (TOR) signalling, both of which have been independently shown to increase lifespan^50, 51^. Curiously, however, there is a large change in transcription during DR^22, 29^. Thus AS-NMD may act as the point of rationalization that leverages increased gene expression to produce newer protein isoforms with diverse function required during DR while increasing mRNA turnover.

The choice of processes that are regulated by AS is commensurate to the needs of metabolic or physiological reprogramming that occur during DR. Genes involved in pyruvate metabolism, tricarboxylic acid cycle, glycolysis and gluconeogenesis as well as oxidative phosphorylation were found to be differentially alternatively spliced. But surprisingly, we did not observe significant enrichment of fatty acid metabolism genes, suggesting that these genes may be regulated transcriptionally. Further, during DR many organisms downregulate fertility but simultaneously upregulate defence systems that preserve the germline for future nutrient-replete conditions^52^. Accordingly, many genes involved in germline and somatic gonad development, embryogenesis and apoptosis were found to undergo AS.

Genes involved in AS, specifically splicing factors, are known to undergo AS-NMD-mediated turnover^53^. Predictably, the genes in RNA processing, splicing and NMD were found to be alternatively spliced, commonly in the worms and hippocampus of mice, apart from signalling pathways, etc. On the other hand, metabolic pathways involving glycolysis, gluconeogenesis, amino-acid metabolism and pentose phosphate pathway undergo AS only in the worms. While the former attests to the conservation of the process and the latter suggests that the AS seen in metabolic genes in worms may represent a whole organism response. Since DR not only ameliorates neurodegenerative diseases but also has positive effect on diseases associated with the kidney, heart and liver^28^, in future it will be interesting to study organ-specific AS in mammals undergoing DR.

The heterogeneous nuclear ribonucleoproteins (hnRNPs) are RNA-binding proteins that form complexes with heterogeneous nuclear RNA. Our reverse genetic screen revealed the role of *hrpu-1* in DR-mediated lifespan extension. *Hrpu-1* belongs to the HNRNPU class of proteins and has some similarity to human HNRNPUL1 and HNRNPUL2 in their SAP (amino acids 18–52, *E*-value 7.85e−06) and SPKY (amino acids 946–1128, *E*-value 3.88e−38) domains. Additionally, it possesses a AAA domain (amino acids 1168–1314, *E*-value 2.62e−26) that uses ATP hydrolysis to bring about conformational changes and exert mechanical force on a substrate^54^. Like other HNRNPs, human HNRNPUL1 is known to play a role in RNA metabolism and transport^55, 56^. However, it has additional role in DNA damage response^57^. In *C. elegans*, RNAi screens have revealed the role of *hrpu-1* in embryo development, germ cell development, lipid storage, locomotion, nematode larval development and regulation of cell proliferation as well as regulation of meiotic nuclear division (http://www.wormbase.org/species/c_elegans/gene/WBGene00012769). Functionally, it is predicted to interact with many other splicing factors^58^. Here we define a new function of *hrpu-1* in worms.

Genes involved in metabolism as well as splicing were found to incorporate introns following AS and were actively degraded by NMD. However, most unexpectedly we found that genes involved in innate immunity were degraded by AS-NMD; these genes showed up when *smg-2* was knocked down in *eat-2(ad1116)*. This was quite interesting as previous studies relate DR to immune function, although the outcomes are quite complex and contradictory. In *C. elegans* that only has the innate arm of immune response, DR does not enhance resistance to the Gram-negative bacteria *Pseudomonas*,^59^ though other pathogens were not checked. Similarly, in *Drosophila* DR has no reported effect on pathogen resistance^60^. On the other hand, rodent studies have shown that, while DR has beneficial role in adaptive immune response, particularly in T cells, it may impair innate immune response in certain cases^61–63^. For example, CR improves mouse survival after *Salmonella typhimurium* infection^64^ and ameliorates experimental colitis in mice^65^. In contrast, a significant decrease in H_2_O_2_, tumour necrosis factor-α, interleukin-6, nitric oxide and signalling events in macrophages as well as impaired natural killer cell function have been reported in mice and rats undergoing CR^66^. In future, more detailed studies need to be performed to determine the universality of the observed AS-NMD-mediated degradation of innate immunity genes during DR.

The FOXA transcription factor PHA-4 plays a pivotal role in DR-mediated longevity. Lifespan extension brought about by several DR regimes require PHA-4^30, 33, 34^. PHA-4 transcriptionally regulates genes important for superoxide or xenobiotic detoxification during DR^30, 33^. It also plays a major regulatory role in controlling gene expression post-transcriptionally. For example, PHA-4 transcriptionally regulates miRNAs that can potentially target majority of the mRNA it helps transcribe^22^. PHA-4 also directly regulates the expression of a chromatin modifier ZFP-1 that modulates the amplitude and duration of its transcriptional targets during DR^43^. Here we present yet another important regulatory function of PHA-4 where it controls the expression of AS-NMD genes during DR. These observations help justify the important role PHA-4 plays in DR-induced lifespan extension. RNA-regulatory mechanisms are becoming increasingly recognized in aging and longevity assurance. While this study was in revision, Heintz et al.^20^ elucidated the role of splicing factor 1 in RNA splicing homeostasis during DR. On the other hand, our study establishes AS-NMD as a conserved mechanism of gene regulation with a positive bearing on lifespan during DR. Together, these studies elucidate how RNA-regulatory processes could play a determining role in lifespan regulation during energy restriction.

## Methods

### Strains

Unless otherwise mentioned, all strains were maintained at 20 °C on standard Nematode Growth Media (NGM) seeded with *Escherichia coli* (OP50). The *E. coli* bacteria were grown overnight in Luria Bertani (LB) media at 37 °C, and 200 or 1000 ul of the culture was seeded on 60 or 90 mm NGM agar plates, respectively. The plates were set at room temperature for 2–3 days to allow the bacteria to grow. Strains used in the study are: N2 Bristol as WT, *rrf-3(pk1426)II*, *eat-2(ad1116)II*, *eat-2(ad465)II*, *eat-2(ad1116)II;rrf-3(pk1426)II*, *eat-2(ad465)II;rrf-3(pk1426)II*, *him-5(e1490)V*, and *smg-2(e2008)I;him-5(e1490)V*.

### Lifespan

Lifespan analysis was performed as described previously^30^. Briefly, NGM RNAi plates (60 mm diameter) were prepared by supplementing NGM with 100 µg/ml ampicillin and 2 mM isopropyl β-d-1-thiogalactopyranoside (IPTG). RNAi bacteria were grown overnight at 37 °C in LB media containing 100 µg/ml ampicillin and 12.5 µg/ml tetracycline. The cultures were diluted next day (1:100 v/v) in LB containing 100 µg/ml ampicillin and grown at 37 °C till an OD_600_ of 0.6 was attained. The bacterial pellets were then resuspended in (1:10 v/v) 1× M9 buffer containing 1 mM IPTG and 100 µg/ml ampicillin. About 200 µl bacterial suspension was plated on the NGM RNAi plates and allowed to dry for 1 day.

Gravid adult hermaphrodite worms were bleached and the eggs were hatched on plates containing the respective RNAi bacteria. When the worms reached gravid adult stage, they were transferred to respective RNAi plates overlaid with 5-fluorodeoxyuridine (FUDR; final concentration of 100 µg/ml)^67^. For post-YA knockdown, the worms were grown on control RNAi containing plates till L4 or YA and then transferred to the respective RNAi. Worms were scored as dead or alive by tapping them with a platinum wire every 2–3 days. Worms that crawled to the sides of the plates were censored. Lifespan graph was plotted with percentage of live worms on *y* axis and the number of days on the *x* axis. Statistical analyses for survival were conducted using Mantel–Cox log-rank test through the OASIS software available at http://sbi.postech.ac.kr/oasis ^68^. Lifespans are expressed as average lifespan ± SEM for all the lifespan experiments. Full data with the number of animals (*n*) are reported in Supplementary Tables 1, 2, 3 and 4.

### bDR lifespan

The bDR assay was performed by preparing *E. coli* (OP50) or RNAi bDR media as described below. Using a platinum loop, *E. coli* OP50 was streaked on a LB agar plate and incubated at 37 °C for 12 h. From this plate, a single colony was inoculated into 200 ml LB in a 2 litre flask and grown at 37 °C for 12 h in an incubator shaker. The bacterial cells were collected after centrifugation at 5,000 rpm, 4 °C for 10 min. The bacterial pellet was then resuspended in S-basal–cholesterol–antibiotics solution (cholesterol 5 µg/ml, carbenicillin 50 µg/ml, tetracycline 1 µg/ml, kanamycin 10 µg/ml) and diluted to the required OD using S-basal–cholesterol–antibiotics solution. Diluted bacterial solutions were kept at 4 °C for a maximum of 2 weeks. For the RNAi bDR assay, control or *hrpu-1* RNAi were streaked on LB agar plates, supplemented with ampicillin (100 µg/ml) and tetracycline (12.5 µg/ml) and was incubated at 37 °C for 12 h. From each plate, a single colony was inoculated into 200 ml LB containing ampicillin (100 µg/ml) in a 2 litre flask and grown at 37 °C for 12 h in an incubator shaker. The bacterial cells were then collected after centrifugation and diluted using S-basal–cholesterol–antibiotics solution supplemented with 2 mM IPTG. Well-fed gravid adult worms were bleached and eggs were then kept on a 90 mm NGM plate seeded with OP50 bacteria. When the worms reached young adult stage, FUDR (100 µg/ml) was added to each plate to arrest progeny development. After about 24 h, worms were transferred to a single well of a 12-well cell-culture plate containing 1 ml (30 worms/well) of S-basal–cholesterol–antibiotics solution with FUDR (100 µg/ml). The plate was kept on a shaker maintained at 20 °C for 1 h to remove adhering bacteria. During this time, appropriately diluted bacterial suspension for the lifespan were added to the 12-well cell culture plates (1 ml solution/well along with FUDR at 100 µg/ml). After 1 h, 10–12 worms/well were moved from the S-basal to the diluted bacterial suspension using a glass pipette connected to a P200 pipette. Worms were moved to fresh bacterial solutions after every 3–4 days at which point they were scored for movement by prodding using a platinum wire. FUDR supplementation in diluted bacterial suspension was stopped after 8 days. Worms that did not respond to gentle prodding with a worm pick were scored as dead and removed; responsive worms were returned to the experiment. During experiments, plates were maintained at 20 °C in an incubator shaker (Innova 42 incubator shaker, New Brunswick Scientific, Edison, NJ, USA) at rotation of 100 rpm. Range of OD used: 3.0, 1.0, 0.5, 0.25, 0.125, and 0.015625. Full data with the number of animals (*n*) are reported in Supplementary Tables 3 and 4. Statistical analyses for survival were conducted using Mantel–Cox log-rank test through the OASIS software available at http://sbi.postech.ac.kr/oasis ^68^ with respect to OD_600_ of 3.0. The response to bDR between *him-5(e1490)* and *smg-2(e2008);him5(e1490)* were compared using two-way analysis of variance (ANOVA).

### RNA isolation and quantitative real-time PCR

Synchronized L1 starved worms were grown till L4–YA stage after which FUDR was added to prevent progeny growth. After about 24 h, worms were collected in M9 buffer and washed thrice and then frozen in Trizol (Invitrogen, USA). For post L4 or YA RNAi treatment (*hrpu-1* RNAi), synchronized L1 worms were grown on control RNAi till L4, following which they were transferred to RNAi treatment plates (with FUDR) and kept for 48 h. The worms in Trizol were passed through a freeze–thaw cycle and lysed by vigorous vortexing. RNA was purified by phenol:chloroform:isoamylalcohol extraction followed by ethanol precipitation. The concentration of the RNA was determined using NanoDrop 2000 (Thermo Scientific, USA). The quality of the ribosomal 28S and 18S on an agarose gel was used as a measure of integrity. cDNA was synthesized using 2.5 μg RNA employing Superscript III Reverse Transcriptase (Invitrogen). Gene expression levels were determined by quantitative real-time PCR (qRT-PCR) using the DyNAmo Flash SYBR Green mastermix (Thermo Scientific) and Realplex PCR system (Eppendorf, USA), according to the manufacturer’s specifications. Relative gene expression was determined after normalizing the data to *actin*. Statistical analysis was performed by unpaired two-tailed *t*-test or two way ANOVA using GraphPad Prism (GraphPad Software, La Jolla, CA, USA).

Semiquantitative RT-PCR validation of AS events were carried out using Taq polymerase (New England Biolabs, USA) and 34–38 cycles of PCR. The PCR products were resolved on 2% agarose gel along with a 100 bp ladder (BR Biochem, New Delhi, India). The uncropped images are provided in Supplementary Fig. 8.

### RNA-Seq and analysis

L1 synchronized worms (in four biological replicates) were grown on OP50- (for comparing WT and *eat-2(ad1116)*) or RNAi-seeded plates (control or *hrpu-1* or *smg-2*) and RNA were isolated as mentioned above. Quality control of RNA was done using Bioanalyzer 2100 RNA 6000 NanoAssay chip (Agilent Technologies, Santa Clara, CA, USA) and RNA above RNA integrity number = 8 was included for the study. The cDNA libraries were subsequently constructed by the TruSeq RNA Library Prep Kit v2 (Illumina Inc., USA). Sequencing (100 bp paired end, 76, 72 or 34 bp single end) was performed using Illumina Genome Analyzer IIx (GA IIx). Imaging, base calling and quality scoring were done as per standard manufacturer’s guidelines (Illumina Inc.). The demultiplexing and conversion of BCL file format reads to FASTQ file format was done with the Illumina-supported CASAVA v1.8.2 software package. The RNA-seq data has been submitted to NCBI with the BioProject ID PRJNA342407. Day 3 RNA-seq data in four biological replicates was downloaded from E-MTAB-4866.

### AS study

Differential AS events were identified based on the fact that these exons, introns and junctions do not follow the global expression trend in the gene. The read counts for exons and introns were obtained using Rsubread package^69^ while for junctions, output of TopHat pipeline was used^70^. These read counts for each term (exon, intron and junctions) were then subjected to TMM normalization as implemented in edgeR package for R^71^. We used limma package for converting read counts of individual term into expression values via voom-function and applied a linear model^72^. Limma was provided two inputs: structure of experiment in the form of a design matrix and the desired statistical tests to be performed on the expression data as a contrast matrix.

The linear model for each term *k* in a particular gene *i* is:

1 $${r_{ki}} = {{\bf{D}}_{{\alpha _j}}} + {\xi _{ki}}$$

where *r* (samples×1) is a vector containing the log_2_ CPM of limma voom function for term *k* for all the samples, ***D*** is the design matrix (samples×conditions), *α*_*j*_ is a vector of effects (conditions×1) and *ξ*_*ki*_ is an error term. In addition, by using a contrast matrix ***C*** (conditions×contrasts), it is possible to interrogate the linear model on a different null hypothesis, *β*_*i*_, by the following equation:

2 $${\beta _i} = {{\bf{C}}^T} + {\alpha _{k\,j}}$$

The empirical Bayes in limma package (*eBayes* function) provided two-tailed *P* values along with moderated *t*-statistics. The two-tailed *P* values were then converted into one-tailed *P* values using the sign of the corresponding *β*_*i*_ coefficients of each term. These *P* values state the statistical significance of the contrast(s) for each term.

Our procedure uses several terms to pin-point each AS event. In case of differential usage of cassette exons, four different terms were utilized together, i.e., reads mapped to the cassette exons, its flanking junctions and the skipping junction that results from the exclusion of the cassette exon. It employs the logic that, if an exon is included into a transcript, then the exon along with its flanking junctions should be expressed, without any expression of the skipping junction and vice versa. Similarly, for intron retention events two terms were considered, i.e., reads that mapped to the intron itself and to the skipping junction, while taking into account that the flanking exons are also expressed.

The *P* value of all the associated terms were then summarized using Irwin–Hall method to get a combined *P* value (Eq*P* values). Let *H*_*e*_ be the set of terms associated with exon *e* that is included into a transcript for a given gene *j*. In this case, the given exon and the flanking junctions can be considered as expressed together. Let *S*_*e*_ be the set of terms when exon *e* is skipped, i.e., the junction in which reads are mapped to regions of the genome both upstream and downstream of exon *e*. If there is a cassette event (the exon is retained in some condition and skipped in the other), the one-tailed *P* value of the terms in *H*_*e*_ will be close to either zero or one and the *P* value of the term in *S*_*e*_ will be close to either one or zero. Summing up these *P* values (written as pvalues in the equations) gives the statistic *x*_*e*_, as follows:

3 $${x_e} = \mathop {\sum}\limits_{k \,\in \,{H_e}} {{\rm {pvalue}}{s_k}} + \mathop {\sum}\limits_{k \,\in \,{S_e}} {\left( {1 - {\rm {pvalue}}{s_k}} \right)} $$

where (*k* ∈ *H*_*e)*_ denotes the terms (exon and flanking junctions) that expressed along with exon *e* and (*k* ∈ S_*e)*_ are the junctions that skip exon *e*. Supplementary Fig. 7A and Equation 4 show an example of a cassette event. In this example, Junc1 and Junc2 are flanking junctions to exon Ex2, while Junc4 and Junc5 skip Ex2. So the sum of one-tailed *P* values (*x*) for Ex2 would be:

4 $${x_{{\rm{Ex}}2}} = \mathop {\sum}\limits_{k \,\in \,\left\{ {{\rm{junc}}1,\,{\rm{Ex}}2,\,{\rm{Junc}}2} \right\}} {{\rm{pvalue}}{s_k}} + \mathop {\sum}\limits_{k \,\in \,\left\{ {{\rm{Junc}}4,\,{\rm{Junc}}5} \right\}} {\left( {1 - {\rm{pvalue}}{s_k}} \right)} $$

Supplementary Fig. 7B and Equation 5 show an example of intron retention event. In this example, Ex1 and Ex2 are flanking exons to intron Int1, while Junc1 and Junc2 skip Int1. So the sum of one-tailed *P* values (*x*) for Int1 would be:

5 $${x_{{\rm{Int1}}}} = \mathop {\sum}\limits_{k \,\in \,\left\{ {\rm{Int1}} \right\}} {{\rm{pvalue}}{s_k}} + \mathop {\sum}\limits_{k \,\in \,\left\{ {{\rm{Junc1}},\,{\rm{junct2}}} \right\}} {\left( {1 - {\rm{pvalue}}{s_k}} \right)} $$

In either of the above cases, since both the expected *P* value and (1−*P* value) follow a uniform (0,1) distribution, if the null hypothesis holds, they will behave as the sum of uniform distributions, i.e., an Irwin–Hall distribution^73^. The Irwin–Hall distribution has a closed form that is piecewise polynomic. If the *X* statistic is smaller than one (most significant *P* values), the corresponding polynomial term of the distribution is:

6 $${f_{{{\rm{X}}^{\left( x \right)}}}} = \frac{1}{{\left( {n - 1} \right)}}.{x^{\left( {n - 1} \right)}},x \le 1$$

where *x* is the summation of the *P* values and 1−*P* values that appears in equation (3). The one-sided probability to obtain a sum of *P* value^overall^ is given by:

7 $$P\,{\rm{valu}}{{\rm{e}}^{{\rm{overall}}}} = 2\mathop {\int}\limits_0^{{x_e}} {{f_x}\left( x \right){\rm{d}}x} = 2\frac{{{x_e}}}{{n!}}$$

where *P* value^overall^ is the summarized *P* value that is used to rank the exons or introns as likely to be differentially alternative spliced. The data of mice RNA-seq (five biological replicates in case of AL5 and CR5; six biological replicates for AL15 and CR15) were downloaded from GSE69952.

### Alternatively spliced junctions

To identify splice junctions using RNA-seq data, we used an already available junction database created by Ramani et al.^4^ as reference for mapping sequencing reads. First, adapter and quality trimming was done with Cutadapt v1.2 using the parameters -m 15 -q 10 —quality-base 30. Quality-trimmed reads were mapped using Bowtie v.0.12.7^74^ with the following parameters --best -v 2 -5 18 -3 18 for 76 cycles run, --best -v 2 -5 16 -3 16 for 72 cycles run, --best -v 2 -5 30 -3 30 for 100 cycles or --best -v 2 for 34 cycles (junction database was trimmed by 3 base pairs from each side for the 34 cycle run). Using these parameters, we maintained a consistent 40 bp overlap with the reference junction database for 72 or 76 or 100 cycle runs and a 34 base overlap for the 34 cycle run. After mapping, the junctions having no expression in either of the data sets under comparison were excluded. To find expression, RPKM was calculated for each junction. For further studying AS, SI was calculated for all the filtered junctions, using the formula:

8 $${\rm{S}}{{\rm{I}}_j} = {{ln}}\left\{ {\frac{{{\rm{RPK}}{{\rm{M}}_{\boldsymbol{j}}}}}{{{\rm{RPK}}{{\rm{M}}_{\boldsymbol{g}}}}}} \right\}{\rm{mut}} - {{ln}}\left\{ {\frac{{{\rm{RPK}}{{\rm{M}}_{\boldsymbol{j}}}}}{{{\rm{RPK}}{{\rm{M}}_{\boldsymbol{g}}}}}} \right\}{\rm{WT}}$$

9 $${\rm{RPK}}{{\rm{M}}_{\boldsymbol{j}}} = \left\{ {\frac{{{\rm{total}}\,{\rm{read}}\,{\rm{count}}}}{{{\rm{mapped}}\,{\rm{reads}}\,\left( {{\rm{million}}} \right){\rm{ \times junction}}\,{\rm{length}}}}} \right\}$$

10 $${\rm{RPK}}{{\rm{M}}_{\boldsymbol{g}}} = \mathop {\sum}\limits_{i = 1}^{i = n} {{\rm{RPK}}{{\rm{M}}_{\boldsymbol{j}}}} $$

11 $${\rm{NI}} = \left\{ {\frac{{{\rm{RPK}}{{\rm{M}}_{\boldsymbol{j}}}}}{{{\rm{RPK}}{{\rm{M}}_{\boldsymbol{g}}}}}} \right\}$$

where SI_*j*_ = SI of junction *j* and NI = normalized intensity of junction.

By using the above formula, a gene is considered to be undergoing differential AS if the relative contribution of each junction towards the total concentration of the gene product (NI) is different under the contrasting conditions, depicted by the value of SI. For testing statistical significance, two-tailed unpaired *t*-test with Sidak–Bonferroni correction was performed on NI, and *P* values were used as the ranking tool. The data were then subjected to several layers of restrictive filtering as follows. The junctions where NI did not differ significantly (*t*-test score > 0.05) between the two groups were omitted from the analysis. The junctions without reads detected above background (reads ≥ 4) in at least half of the samples (in either condition) were also omitted. Only genes expressed in both sample groups (>5 reads) were included in the analysis. The SI was calculated and SI cutoff was set at <−1.0 or >1.0 in ln scale. The junctions fulfilling all the above filtering criteria were ranked based on their *P* values and manually inspected for validation. To show differential AS in different genes, the spread in the expression pattern of two junctions having most significant SI were plotted as a heat map using MeV v.4.9.

### Intron expression analysis

To find out the expression pattern of introns, first adapter and quality trimming was done with Cutadapt v1.2 using the parameters -m 15 -q 10 —quality-base 30. Quality-filtered reads of replicates were pooled together and were aligned to the *C. elegans* annotated reference genome (WS231) using Bowtie v.0.12.7 with parameters: -q -best -n 0; thus only those reads having 100% mapping were considered for further analysis. Start or End positions of all the introns of *C. elegans* were fetched from Ensemble Genome Browser BioMart Utility, and perl script was used to find the number of reads present within each intron. Introns having <5 reads in all the four conditions were not considered for further analysis. Expression for these filtered introns was found out using RPKM method and fold changes were considered based on the ratio of *smg-2* knockdown over control condition. We made use of UCSC genome browser for visualization of aligned data in.bed format.

### Domain enrichment in differentially expressed exons

Expression pattern for individual exon was calculated using similar pipeline as done for introns. Exons showing a differential AS and whose usage increased in *eat-2(ad1116)* were only considered for domain enrichment analysis. Only exons having an expression (with minimum reads as 5) in *eat-2(ad1116)* were considered. Corresponding exon sequences were fetched from ensemble and batch web CD search tool of ‘CDD: NCBI’s conserved domain database’,^75^ was utilized to obtain the domains. Frequency enrichment of all the domains was calculated. Similarly, three random sets of exons were used as a control and average frequency was calculated to help in comparison.

### GO analysis

All the enrichment of the conserved gene sets was determined using the functional annotation tool in the Database for Annotation, Visualization and Integrated Discovery^76^. Default parameters were taken and the cutoff values of *P* value ≤ 0.05 (Fisher Exact Test) and/or false discovery rate at ≤10.0 were used. For functional analysis, GO (GO_BP_Direct), KEGG Pathway and Interpro Domains were used. The raw values of different GO results are reported in Supplementary Data 5.

### ChIP-seq data analysis

The ChIP-seq data analysis was performed using parameters mentioned in our previous publication^43^. PHA-4 ChIP-seq data for L3 stage (SRA-NCBI GSE50301) were downloaded from MODENCODE (http://www.modencode.org/) in .sra format. Downloaded data were converted into FASTQ format using NCBI-recommended SRA toolkit (version 2.2.2a). Converted FASTQ of replicates were merged and used for further analysis. Reads were aligned to the *C. elegans* genome (WS230) using Bowtie (v0.12.7)^74^ with the following parameters: -q -m 1 --best --strata. Mapped reads were used for peak calling and calculation of read density. Enriched peaks were identified using the peak calling algorithm MACS (v1.4.2)^77^ using the following parameters: --mfold = 5,30 --bw = 175 -w. Statistically significant peaks (*P* < 1×10^−5^) were used for further analysis. To find target genes, PeakAnalyzer (v1.4)^78^ program was used and all genes having peaks within 2 kb of the promoter region were considered for further analysis. UCSC genome browser^79^ was used for visualizing aligned data as wig files.

### Code availability

The code can be accessed from GitHub using the following link, https://github.com/ravidattasharma/EP-IPrnaSeq.

### Data availability

All the RNA-seq data related to this study have been deposited in SRA with the accession code SRP089617. All other data are available from the corresponding author upon reasonable request.

## Electronic supplementary material

Supplementary Information

Supplementary Data 1

Supplementary Data 2

Supplementary Data 3

Supplementary Data 4

Supplementary Data 5

Supplementary Data 6

Supplementary Data 7

Supplementary Data 8

Supplementary Data 9

Supplementary Data 10

Supplementary Data 11

Supplementary Data 12
