# Supplementary material for: Differential alternative splicing coupled to nonsense-mediated decay of mRNA ensures dietary restriction-induced longevity
Source: Nat Commun. 2017 Aug 21;8:306. doi: 10.1038/s41467-017-00370-5 (PMC5563511; doi:10.1038/s41467-017-00370-5)
Supplement: Supplementary file 1 — Supplementary Information [file 41467_2017_370_MOESM1_ESM.pdf]

File Name: Supplementary Information
Description: Supplementary Figures and Supplementary Tables

File Name: Supplementary Data 1
Description: Differential exon usage in *eat-2(ad1116)* as shown in Figure 1A, 1B.

File Name: Supplementary Data 2
Description: Differential intron retention in *eat-2(ad1116)* as shown in Figure 1A, 1B.

File Name: Supplementary Data 3
Description: Alternatively spliced junctions in *eat-2(ad1116)* on day 3, as determined by splicing index and shown in Figure 1D.

File Name: Supplementary Data 4
Description: Alternatively spliced junctions in *eat-2(ad1116)* on day 1, as determined by splicing index for Supplementary Figure 1B.

File Name: Supplementary Data 5
Description: Function of genes determined by Gene Ontology, KEGG pathway and Domain analysis.

File Name: Supplementary Data 6
Description: Domains encoded by exons whose usages are increased in *eat-2(ad1116)* compared to WT as shown in Supplementary Figure 1D.

File Name: Supplementary Data 7
Description: Differential exon usage in caloric restricted mice (5 months).

File Name: Supplementary Data 8
Description: Differential intron retention in caloric restricted mice (5 months).

File Name: Supplementary Data 9
Description: Differential exon usage in caloric restricted mice (15 months).

File Name: Supplementary Data 10
Description: Differential intron retention in caloric restricted mice (15 months).

File Name: Supplementary Data 11
Description: Comparison of alternatively spliced junctions between WT and *eat-2(ad1116)* on control RNAi with that between *eat-2(ad1116)* on control and *hrpu-1* RNAi.

File Name: Supplementary Data 12
Description: Intron retention when *smg-2* is knocked down in WT or *eat-2(ad1116).*

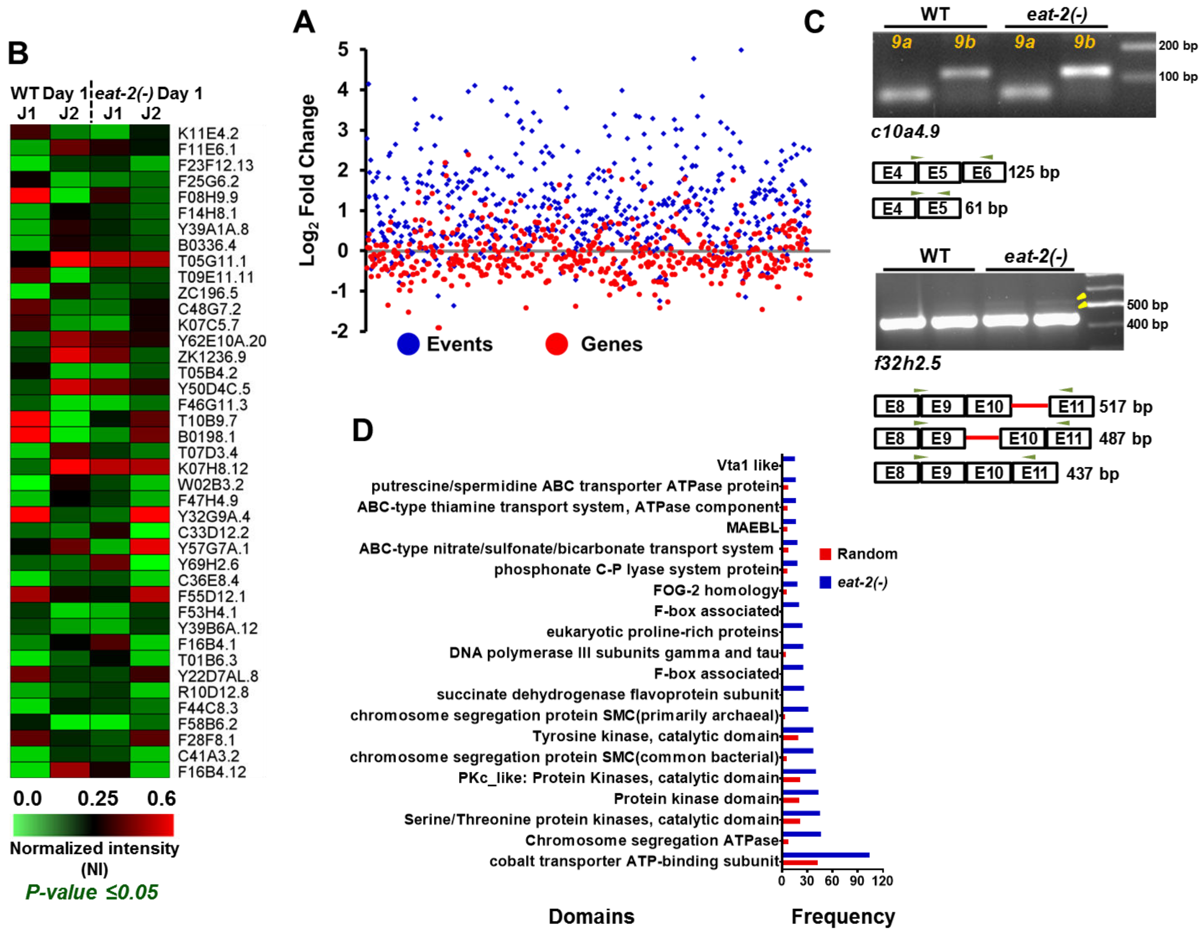

Supplementary Figure 1. **(A)** Scatter plot showing differential expression of AS events in relation to the expression of the corresponding gene. The relative RPKM of an AS event [*eat-2(ad1116) vs* WT] and the corresponding gene in which that event takes place is plotted. **(B)** Heat map depicting differential AS of top 41 genes in day 1 *eat-2(ad1116)* [represented as *eat-2(-)*]worms. J1 and J2 indicate two junctions of the same gene showing most significant differences in their splicing indices. Statistical analysis was performed using two-tailed unpaired *t*-test with Sidak-Bonferroni correction for individual events. **(C)** Validation of AS events from Day 1 *eat-2(ad1116)* samples. In case of *c10a4.9*, the isoform having exon 6 is increased in *eat-2(ad1116).* In *f32h2.5*, two other isoforms (indicated with yellow arrows) that retain introns appear in *eat-2(ad1116).* For *f32h2.5*, the two lanes for each sample are biological replicates. In *c10a4.9,* primer pairs 9a and 9b detects two different isoforms. Similar results were observed in at least 3 biological replicates. **(D)** Appearance of specific protein domains in the differentially spliced exons. The 1010 exons, whose usage increased in *eat-2(ad1116)* compared to WT at day 1, were translated and searched using NCBI Web CD-BLAST to identify the domains present (blue bars). Frequency indicates the total number of hits for the particular domain. Three sets of 1000 random exons were also translated and searched as a control exercise; the average frequency is plotted (red bars).

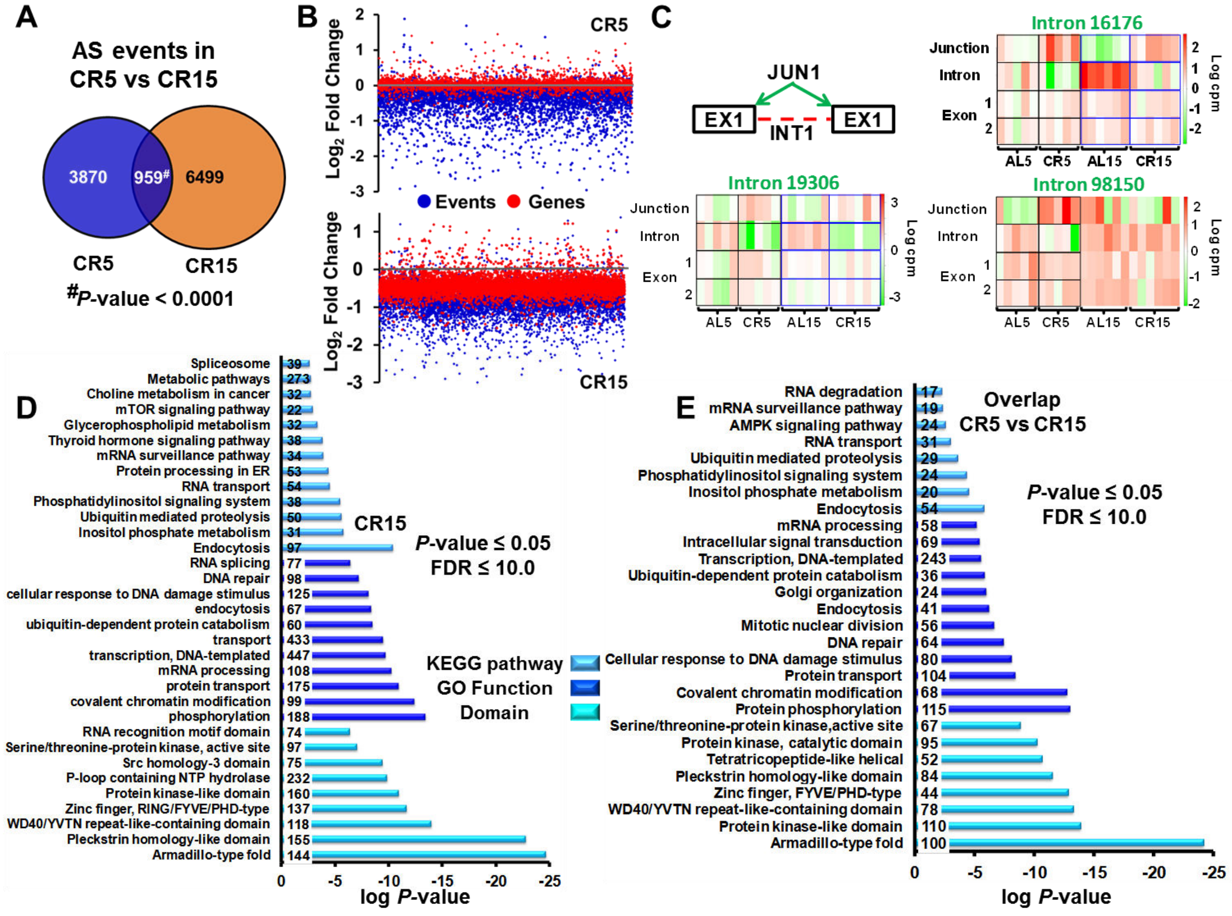

Supplementary Figure 2. **(A)** Significant overlap between AS events during early (CR5) and late (CR15) phases of CR. *P*-value determined by hypergeometric test. **(B)** Scatter plot showing differential expression of AS events in relation to the expression of the corresponding gene. The relative RPKM of an AS event [CR *vs* AL] and the corresponding gene in which that event takes place is plotted. CR- Caloric Restriction; AL- *ad libitum*. **(C)** Representative images of AS involving intron retention. For this, two terms were considered, reads mapping to the introns (INT1) and that of the skipping junction when the intron is not included (JUN1; see cartoon), while ensuring that flanking exons are expressed. Each box represents 5 (for CR5 or AL5) or 6 (for CR15 or AL15) biological replicates in the RNA-seq experiments. In case of Intron 19306 and 16176, the read counts mapping to the intron or the junction were reciprocal in both AL5 vs CR5 as well as in AL15 vs CR 15. In Intron 98150, the same was observed only in case of AL5 vs CR5. **(D)** KEGG pathway, Gene ontology term enrichment and protein domains was determined by DAVID Functional Annotation tool using differentially AS genes in mice undergoing CR for 15 months. The listed pathways have a *P*-value less than 0.05 (Fisher Exact Test) and FDR less than 10.0. The numbers within the blue bars represent genes detected in the category. **(E)** The above analysis was also performed using genes that were showing differential AS in both CR5 and CR15.

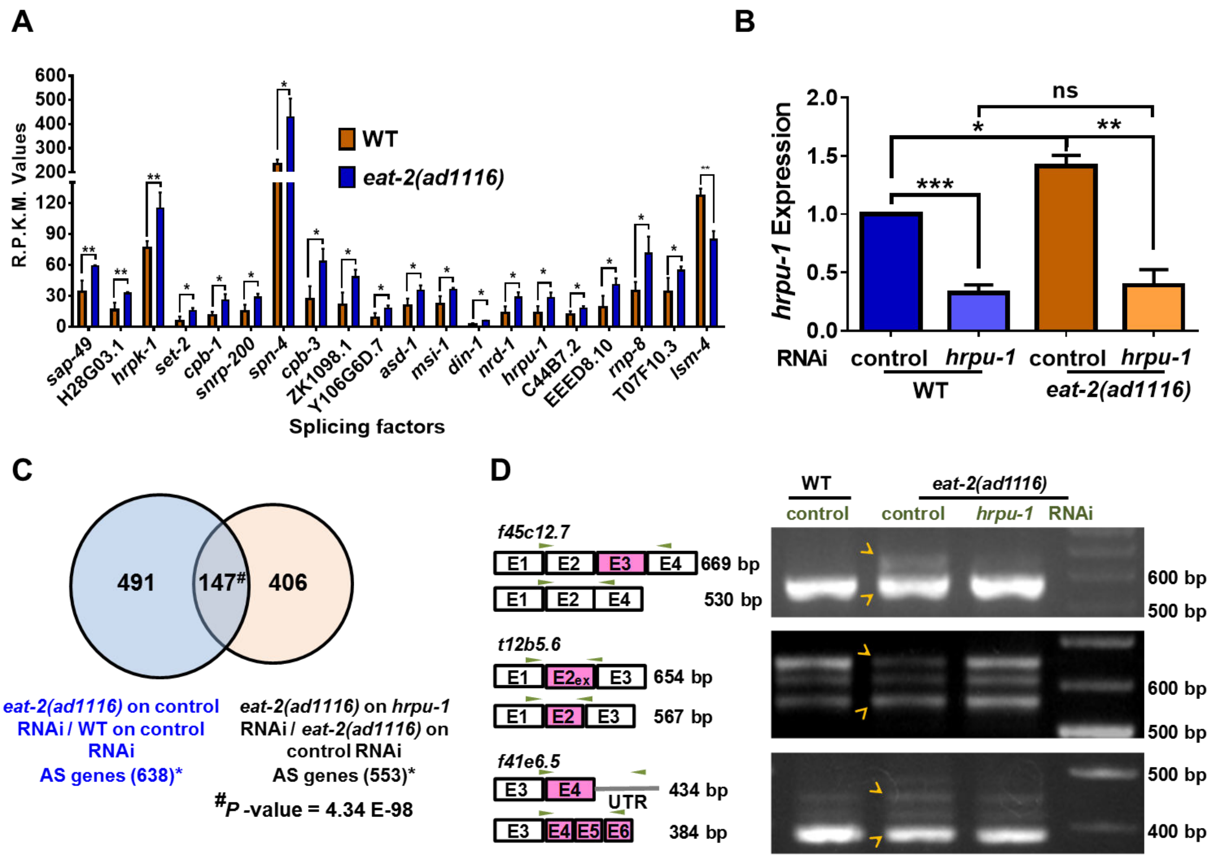

Supplementary Figure 3. **(A)** Upregulation of splicing factors as determined by RNA-seq analysis comparing average RPKM of *eat-2(ad1116)* to WT on day 1 in two biological replicates using CLC Genomics Workbench (Qiagen Bioinformatics, USA). (Error bars = SEM). Only the genes with significant changes in expression are shown. *P*-value ≤ 0.05, Kal's-Z test. **(B)** Determination of knockdown efficiency of *hrpu-1* RNAi. WT or *eat-2(ad1116)* was grown on control or *hrpu-1* RNAi (initiated at L4 and harvested on day 3 of adulthood) and QRT-PCR was used to quantify the expression of *hrpu-1* transcript. Average of 3 biological replicates shown. Statistically analysis was performed using unpaired two-tailed *t*-test, **P* ≤ 0.05; ***P*≤ 0.01; ****P*≤ 0.001; ns= non-significant). **(C)** Overlap of genes that have increased AS in *eat-2(ad1116)* compared to WT, with those controlled by *hrpu-1* in *eat-2(ad1116)*. *P*-value determined by hypergeometric test. **(D)** Validation of a few AS events regulated in *eat-2(ad1116)* in a *hrpu-1*-dependent manner. In case of *f45c12.7*, the isoform containing exon 3 was included in *eat-2(ad1116)* but was suppressed when *hrpu-1* was knocked down. The relative use of isoforms in *t12b5.6* changes in *eat-2(ad1116)* compared to WT, but reverts when *hrpu-1* is knocked down. In *f41e6.5*, the isoform having the UTR is preferred in *eat-2(ad1116)*, in a *hrpu-1*-dependent manner. The experiments were repeated three times. (Error bars = SEM).

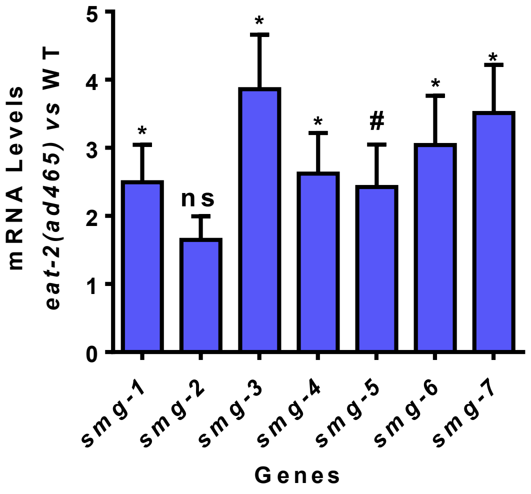

Supplementary Figure 4. QRT-PCR showing that the genes of NMD pathway are upregulated in *eat-2(ad465)*. Expression levels were normalized to actin and compared to Day 1 of WT worms. Averages of four biological replicates are shown (Error bars = SEM). Asterisks indicate statistically significant differences to the corresponding WT samples, as calculated by unpaired two-tailed *t*-test (* $P \leq 0.05$; # $P = 0.06$; ns= non-significant).

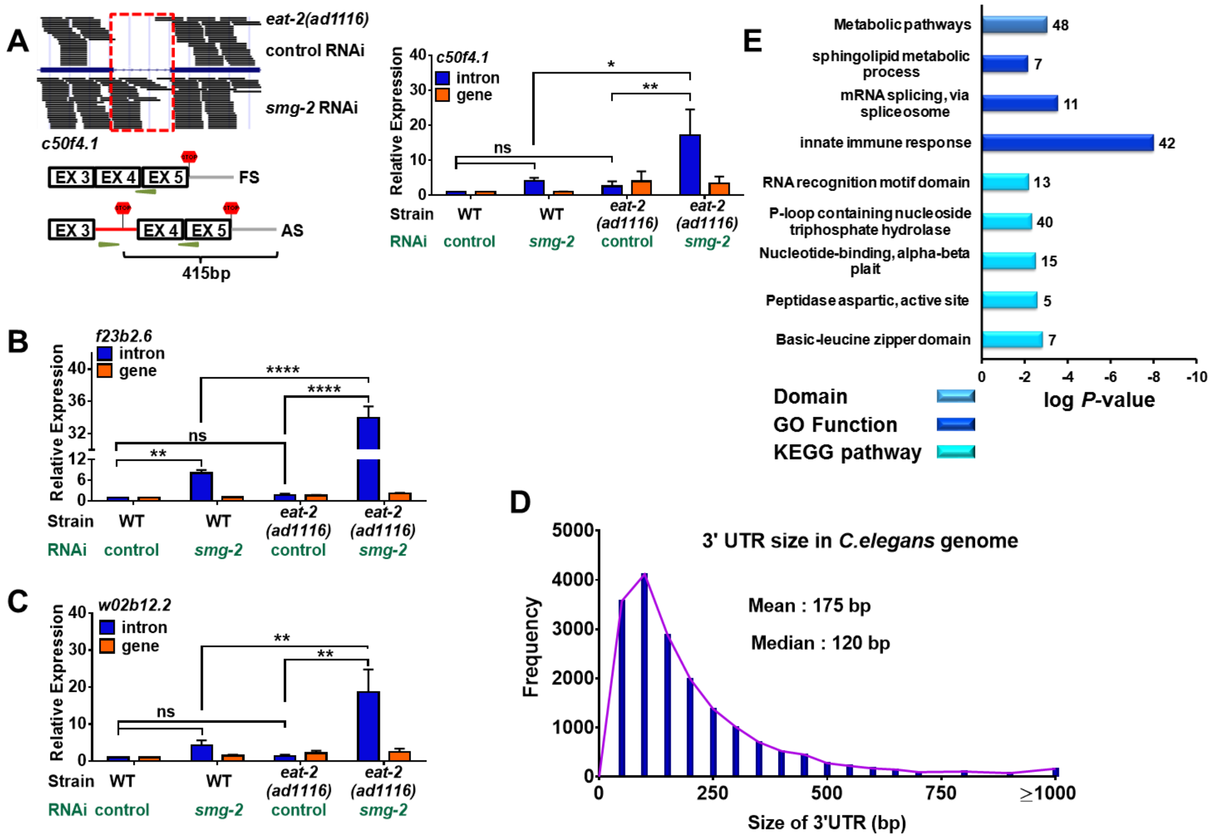

Supplementary Figure 5. (A) UCSC Browser views show parts of the *c50f4.1* gene and mapped sequencing reads when *eat-2(ad1116)* worms were grown on control or *smg-2* RNAi (upper panel). A graphical representation (GR) of the two alternate forms (FS-functionally spliced with normal stop codon; AS-alternatively spliced with a PTC) of the transcripts is shown below the browser view. AS product with PTC is marked in the GR. Primer pairs were designed to quantify the expression of the intron-containing transcript. Distance between PTC and the start of poly-A site is indicated. The QRT-PCR validation of the events, along with the expression of the genes, is presented on the right-hand side. Differential intron retention (blue bars) was detected in *eat-2(ad1116)* and WT, grown on *smg-2* or control RNAi. Expression levels were normalized to actin and compared to WT on control RNAi. Averages of three biological replicates are shown (error bars = SEM). Asterisks indicate statistically significant differences as calculated using unpaired two-tailed *t*-test (* $P \leq 0.05$; ** $P \leq 0.01$, ***$P \leq 0.001$ and **** $P \leq 0.0001$; ns- non-significant). The changes in expression of the respective genes (orange bars) were ns. **(B-C)** Quantification of differential intron retention in *f23b2.6* and *w02b12.2* in WT and *eat-2(ad1116)* grown on control or *smg-2* RNAi. Details same as above. **(D)** Frequency distribution of the distance of the normal stop codons to the 3'-end of all the transcripts with annotated 3' UTR in *C. elegans*. **(E)** Functional annotation of the genes corresponding to the differentially expressed introns in *eat-2 (ad1116)* upon NMD knock down.

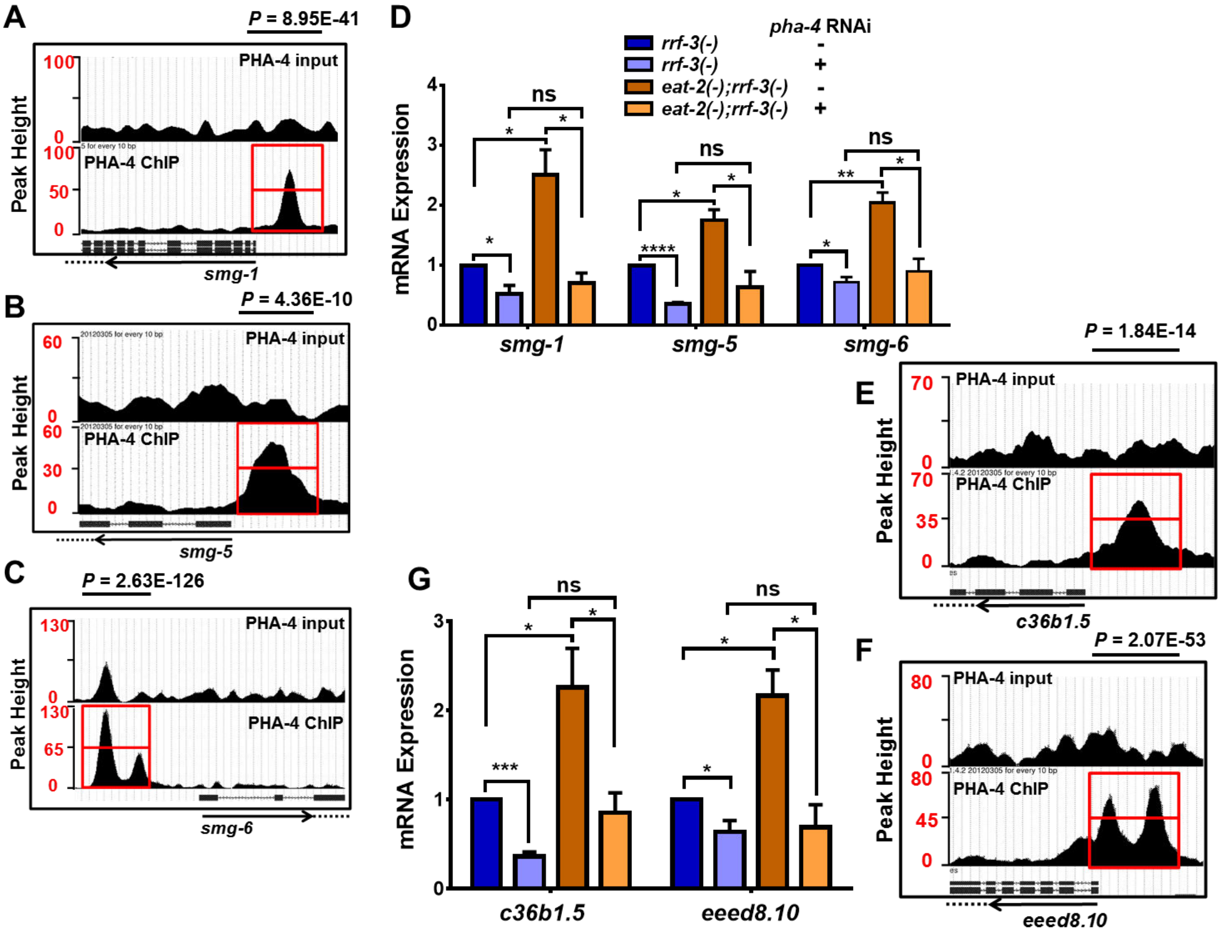

Supplementary Figure 6. (A-C) UCSC browser view of PHA-4/FOXA peaks on *smg-1*, *smg-5* and *smg-6* promoters or **(E-F)** on *c36b1.5* and *eeed8.10* promoters, as determined by ChIP-seq analysis of *unc-119(ed3) III; wgIs37* (*OP37*) strain; data mined from MODENCODE and reanalysed using our bioinformatic pipeline. Red boxes indicate the promoter regions where peaks are observed. Upper panel shows peaks in input samples. **(D, G)** qRT-PCR showing that (D) NMD genes *smg-1*, *smg-5* and *smg-6* or (G) splicing mediator *c36b1.5* and *eeed8.10* are upregulated in *eat-2(ad465)* in a *pha-4*-dependent manner. Expression levels were normalized to actin. Averages of three biological replicates are shown (Error bars = SEM). Asterisks indicate statistically significant differences, as calculated by unpaired two-tailed *t*-test (* $P \leq 0.05$; ** $P \leq 0.01$, *** $P \leq 0.001$, **** $P \leq 0.0001$, ns= non-significant). The + sign indicates presence of *pha-4* RNAi while - indicates control (vector) RNAi.

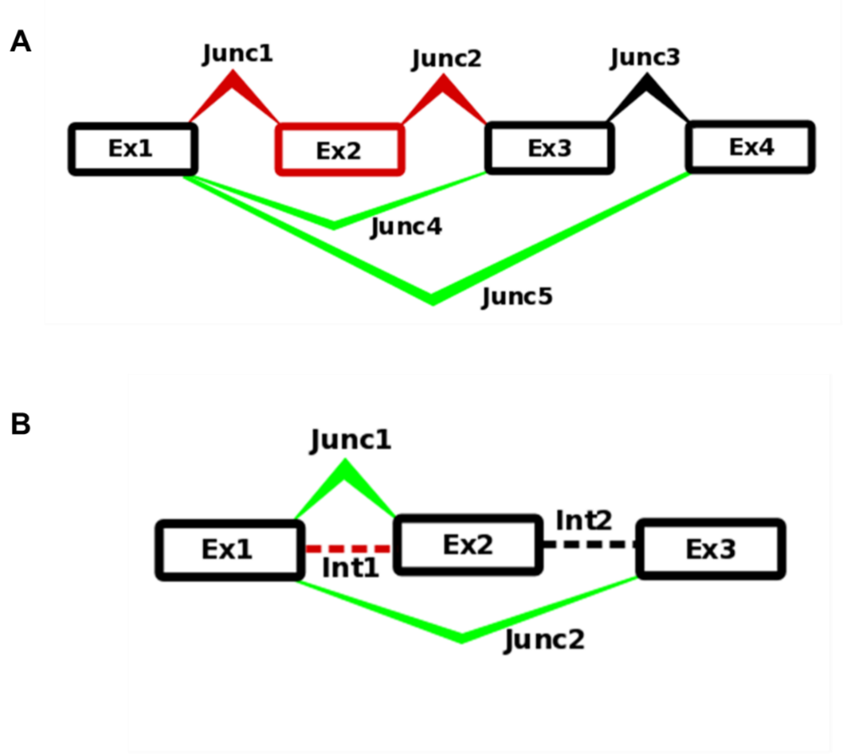

Supplementary Figure 7. **(A)** Example of a cassette exon event. *Junc1* and *Junc2* are flanking junctions to exon *Ex2*, while *Junc4* and *Junc5* skip *Ex2*. **(B)** Example of an intron retention event. Int1 denotes intron that is retained. *Ex1* and *Ex2* are flanking exons to intron *Int1*, while *Junc1* and *Junc2* skip *Int1*.

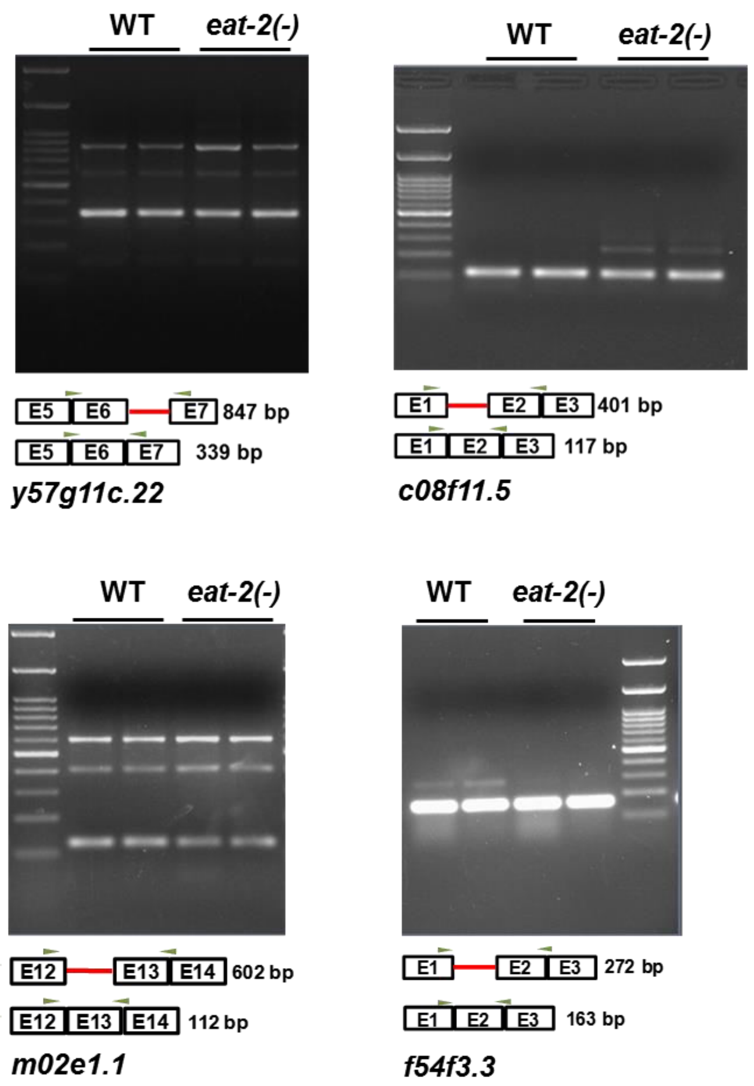

Supplementary Figure 8. Uncropped gel images for Figures 1C.

Supplementary Table 1: RNAi screen to determine the effects of knocking down splicing mediators on WT or *eat-2(ad1116)* life spans

Genotype	RNAi	n	Mean LS ± SEM (Days)	% Change w.r.t. Control RNAi	*P-* value (control Vs RNAi)
rrf-3(pk1426)					
	control	174	16.89 ± 0.24		
	c44b7.2	172	19.6 ± 0.27	16.04	<0.0001
	f46a9.6	163	19 ± 0.23	12.49	<0.0001
	h28g03.1	129	19.08 ± 0.3	12.97	<0.0001
	f53g2.6	141	19.18 ± 0.34	13.56	<0.0001
	y41e3.11	121	17.08 ± 0.22	1.12	0.0351
	c33h5.12	173	17.23 ± 0.24	2.01	0.482
	f26b1.2	130	16.24 ± 0.22	-3.85	0.0012
	r05d11.7	142	16.21 ± 0.21	-4.03	0.0017
	cc4.3	147	16.17 ± 0.24	-4.26	0.5023
	c25a1.4	126	16.76 ± 0.24	-0.77	0.0486
	f28d9.1	165	16.54 ± 0.19	-2.07	0.0039
	eeed8.7	168	18.05 ± 0.17	6.87	0.0278
	y57g11a.5	148	17.26 ± 0.18	2.19	0.8303
	y49f6b.4	133	17.95 ± 0.2	6.28	0.0065
	y116a8c.32	141	16.21 ± 0.18	-4.03	0.038
	c18d11.4	107	15.81 ± 0.22	-6.39	0.0026
	r10e9.1	99	16.88 ± 0.16	-0.06	0.0552
	f42a6.7	132	19.44 ± 0.19	15.10	<0.0001
	y47d3a.27	116	17.35 ± 0.19	2.72	0.6792
	w02b12.2	95	17.48 ± 0.17	3.49	0.622
	y116a8c.35	99	16.88 ± 0.16	-0.06	0.0552
	y111b2a.18	69	16.91 ± 0.19	0.12	0.0357
eat-2(ad1116);rrf-3(pk1426)					
	control	125	27.84 ± 0.46		
	c44b7.2	89	28.82 ± 0.61	3.52	0.1182
	f46a9.6	129	28.31 ± 0.5	1.69	0.3586

	h28g03.1	118	28.23 ± 0.66	1.40	0.1086
	f53g2.6	73	27.05 ± 0.62	-2.84	0.3337
	y41e3.11	170	17.08 ± 0.14	-38.65	<0.0001
	c33h5.12	148	26.95 ± 0.41	-3.20	0.138
	f26b1.2	131	29.78 ± 0.54	6.97	0.0014
	r05d11.7	150	28.42 ± 0.35	2.08	0.7137
	cc4.3	162	26.86 ± 0.48	-3.52	0.5135
	c25a1.4	101	32.82 ± 0.67	17.89	<0.0001
	f28d9.1	100	26.06 ± 0.67	-6.39	0.2178
	eeed8.7	131	31.17 ± 0.62	11.96	<0.0001
	y57g11a.5	117	28.68 ± 0.72	3.02	0.0227
	y49f6b.4	118	24.31 ± 0.51	-12.68	<0.0001
	y116a8c.32	122	26.1 ± 0.57	-6.25	0.0743
	c18d11.4	114	27.38 ± 0.5	-1.65	0.5203
	r10e9.1	94	25.95 ± 0.61	-6.79	0.3138
	f42a6.7	29	34.21 ± 1.25	22.88	<0.0001
	y47d3a.27	118	32.09 ± 0.65	15.27	<0.0001
	w02b12.2	69	30.28 ± 0.56	8.76	0.0013
	y116a8c.35	96	24.73 ± 0.49	-11.17	<0.0001
	y111b2a.18	95	30.94 ± 0.58	11.14	<0.0001

**Genotype**	**RNAi**	**n**	**Mean LS ± SEM (Days)**	**% Change w.r.t. Control RNAi**	***P*- value (control Vs RNAi)**
rrf-3(pk1426)					
	control	101	19.02 ± 0.28		
	c18e3.5	88	18.14 ± 0.27	-4.63	0.5749
	b0414.5	97	18.27 ± 0.32	-3.94	0.0632
	c17e4.5	111	15.16 ± 0.06	-20.29	<0.0001
	r06c1.4	135	20.46 ± 0.32	7.57	0.0006
	eeed8.12	84	19.05 ± 0.35	0.16	0.8069
	f40f8.9	52	17.87 ± 0.46	-6.05	0.3079
	f07a11.6	69	17.81 ± 0.21	-6.36	0.0006
	r74.5	69	17.81 ± 0.21	-6.36	0.0006
	c40h1.1	84	18.14 ± 0.31	-4.63	0.5287
	t12d8.2	110	18.72 ± 0.3	-1.58	0.2023

	c02b10.4	69	17.81 ± 0.21	-6.36	0.0006
	k07h8.10	60	18.95 ± 0.31	-0.37	0.0054
	f08g12.2	111	16.91 ± 0.25	-11.09	<0.0001
	c27h5.3	77	20.27 ± 0.27	6.57	0.5571
	eeed8.10	128	19.02 ± 0.18	0.00	0.0001
	t08b6.5	89	19.55 ± 0.18	2.79	0.0088
	c26e6.9	29	17.97 ± 0.32	-5.52	0.025
	c36b1.5	152	17.16 ± 0.21	-9.78	<0.0001
	c44e4.4	96	18.92 ± 0.29	-0.53	0.7484
	c49h3.5	46	17.35 ± 0.32	-8.78	0.0003
	c50d2.5	136	18.65 ± 0.21	-1.95	0.1678
	f11a10.2	67	18.13 ± 0.22	-4.68	<0.0001
	f28f8.3	79	20.97 ± 0.44	10.25	0.028
	f29c4.7	47	19.96 ± 0.49	4.94	0.0864
	f32a5.7	72	18.07 ± 0.31	-4.99	0.2057
	f44g4.4	87	20.34 ± 0.26	6.94	<0.0001
	k02f3.11	125	18.38 ± 0.27	-3.36	0.6525
	k08d10.3	135	18.72 ± 0.26	-1.58	0.4792
	k08d10.4	110	18.96 ± 0.29	-0.32	0.9324
	m03c11.7	31	16.71 ± 0.59	-12.15	<0.0001
	r05d3.8	78	23.01 ± 0.78	20.98	<0.0001
	r07e5.14	60	19.5 ± 0.36	2.52	0.0139
	t04a8.6	75	17.51 ± 0.39	-7.94	<0.0001
	t10b11.3	127	19.41 ± 0.28	2.05	0.33
	t10g3.6	113	18.58 ± 0.23	-2.31	0.0131
	w07e6.4	81	12.3 ± 0.2	-35.33	<0.0001
	y106g6d.7	40	17.75 ± 0.34	-6.68	<0.0001
	y106g6h.2	86	17.31 ± 0.31	-8.99	0.0001
eat-2(ad1116);rrf-3(pk1426)	control	176	34.27 ± 0.52		
	c18e3.5	93	33.28 ± 0.65	-2.89	0.1633
	b0414.5	157	30.52 ± 0.42	-10.94	<0.0001
	c17e4.5	117	28.88 ± 0.53	-15.73	<0.0001
	r06c1.4	67	33.64 ± 0.88	-1.84	0.735
	eeed8.12	89	30 ± 0.56	-12.46	<0.0001
	f40f8.9	107	31.17 ± 0.54	-9.05	<0.0001

f07a11.6	137	28.05 ± 0.45	-18.15	<0.0001
r74.5	91	29.53 ± 0.57	-13.83	<0.0001
c40h1.1	166	34.27 ± 0.26	0.00	1
t12d8.2	115	30.01 ± 0.56	-12.43	<0.0001
c02b10.4	99	28.84 ± 0.41	-15.84	<0.0001
k07h8.10	165	31.33 ± 0.56	-8.58	0.0001
f08g12.2	118	31.14 ± 0.7	-9.13	0.0003
c27h5.3	142	28.04 ± 0.48	-18.18	<0.0001
eeed8.10	138	27.7 ± 0.46	-19.17	<0.0001
t08b6.5	160	26.84 ± 0.39	-21.68	<0.0001
c26e6.9	166	23.9 ± 0.27	-30.26	<0.0001
c36b1.5	141	18.26 ± 0.18	-46.72	<0.0001
c44e4.4	43	28.19 ± 0.74	-17.74	<0.0001
c49h3.5	103	29.81 ± 0.55	-13.01	<0.0001
c50d2.5	123	18.37 ± 0.12	-46.40	<0.0001
f11a10.2	87	26.98 ± 0.36	-21.27	<0.0001
f28f8.3	168	24.35 ± 0.32	-28.95	<0.0001
f29c4.7	80	26.11 ± 0.45	-23.81	<0.0001
f32a5.7	153	26.73 ± 0.27	-22.00	<0.0001
f44g4.4	62	23.55 ± 0.47	-31.28	<0.0001
k02f3.11	96	28.13 ± 0.54	-17.92	<0.0001
k08d10.3	118	23.98 ± 0.4	-30.03	<0.0001
k08d10.4	92	26.67 ± 0.49	-22.18	<0.0001
m03c11.7	61	27.38 ± 0.63	-20.11	<0.0001
r05d3.8	137	23.8 ± 0.42	-30.55	<0.0001
r07e5.14	75	29.05 ± 0.54	-15.23	<0.0001
t04a8.6	133	29.54 ± 0.42	-13.80	<0.0001
t10b11.3	96	20.51 ± 0.28	-40.15	<0.0001
t10g3.6	129	21.09 ± 0.25	-38.46	<0.0001
w07e6.4	78	20.9 ± 0.5	-39.01	<0.0001
y106g6d.7	80	26.31 ± 0.45	-23.23	<0.0001
y106g6h.2	61	24.23 ± 0.48	-29.30	<0.0001

Genotype	RNAi	n	Mean LS ± SEM (Days)	% Change w.r.t. Control RNAi	*P*- value (control Vs RNAi)
rrf-3(pk1426)	control	93	17.22 ± 0.31		
	r09b3.2	104	18.55 ± 0.26	7.72	0.0807
	k08f4.2	51	18.18 ± 0.32	5.57	0.8394
	y116a8c.42	58	16.59 ± 0.35	-3.66	0.1734
	zc404.8	88	19.43 ± 0.36	12.83	0.000026
	zk863.7	86	19.43 ± 0.48	12.83	0.0001
	c52e4.3	59	15.37 ± 0.35	-10.74	0.0002
	t07f10.3	22	18.32 ± 0.47	6.39	0.9661
	t01d1.2	44	13.82 ± 0.24	-19.74	<0.0001
	f09d1.1	29	17.59 ± 0.47	2.15	0.4642
	c30b5.4	78	18.63 ± 0.35	8.19	0.0681
	t13h5.4	105	18.65 ± 0.23	8.30	0.0003
	r05h10.2	75	18.57 ± 0.33	7.84	0.1382
	zk652.1	69	18.91 ± 0.5	9.81	0.0005
	c50c3.6	89	17.09 ± 0.32	-0.75	0.346
	zk1098.1	99	18 ± 0.2	4.53	0.8759
	b0035.12	46	17 ± 0.33	-1.28	0.0245
	f19f10.9	31	13.19 ± 0.36	-23.40	<0.0001
	f32b4.4	128	18.09 ± 0.17	5.05	0.3732
	t23f6.4	48	19.38 ± 0.53	12.54	0.0245
	w04d2.6	67	17.3 ± 0.13	0.46	0.1247
	y49e10.15	66	16.12 ± 0.23	-6.39	0.0087
	f56a8.6	117	18.9 ± 0.2	9.54	<0.0001
	w08e3.1	118	17.61 ± 0.3	2.26	0.9547
eat-2(ad1116);rrf-3(pk1426)	control	81	25.23 ± 0.44		
	r09b3.2	198	30.83 ± 0.42	22.20	0
	k08f4.2	86	24.77 ± 0.32	-1.82	0.1592
	y116a8c.42	96	22.89 ± 0.25	-9.27	<0.0001
	zc404.8	160	32.84 ± 0.45	30.16	<0.0001
	zk863.7	63	25.56 ± 0.5	1.31	0.9105
	c52e4.3	113	24.06 ± 0.28	-4.64	0.0057

t07f10.3	123	25.89 ± 0.47	2.62	0.0229
t01d1.2	166	29.34 ± 0.56	16.29	<0.0001
f09d1.1	123	28.36 ± 0.64	12.41	<0.0001
c30b5.4	135	30.31 ± 0.62	20.13	<0.0001
t13h5.4	124	31.99 ± 0.58	26.79	<0.0001
r05h10.2	183	31.49 ± 0.5	24.81	<0.0001
zk652.1	193	28.53 ± 0.38	13.08	<0.0001
c50c3.6	192	28.7 ± 0.46	13.75	<0.0001
zk1098.1	190	25.53 ± 0.34	1.19	0.098
b0035.12	128	23.82 ± 0.32	-5.59	0.0077
f19f10.9	78	20.9 ± 0.5	-17.16	<0.0001
f32b4.4	21	15.29 ± 0.19	-39.40	<0.0001
t23f6.4	137	23.8 ± 0.42	-5.67	0.5983
w04d2.6	50	20.64 ± 0.29	-18.19	<0.0001
y49e10.15	111	20.95 ± 0.35	-16.96	<0.0001
f56a8.6	179	24.08 ± 0.31	-5.17	0.0212
w08e3.1	174	22.26 ± 0.48	-11.77	0.0407

Supplementary Table 2: Representative data of life span analysis after knocking down splicing mediators. Two alleles of *eat-2* mutant were used.

Experiment 1

Genotype	RNAi	n	Mean LS ± SEM (Days)	% Change w.r.t. Control RNAi	*P*- value (control Vs RNAi)	Censored population
rrf-3(pk1426)	control	191	17.76 ± 0.15			7
	b0035.12	81	16.58 ± 0.29	-6.64	0.0006	3
eat-2(ad1116);rrf-3(pk1426)	control	108	30.19 ± 0.59			5
	b0035.12	88	20.17 ± 0.23	-33.19	<0.0001	4
eat-2(ad465);rrf-3(pk1426)	control	226	29.27 ± 0.28			9
	b0035.12	110	18.87 ± 0.27	-35.53	<0.0001	2
rrf-3(pk1426)	control	191	17.76 ± 0.15			7
	c36b1.5	102	16 ± 0.2	-9.91	<0.0001	2
eat-2(ad1116);rrf-3(pk1426)	control	108	30.19 ± 0.59			5
	c36b1.5	119	18.56 ± 0.24	-38.52	<0.0001	6
eat-2(ad465);rrf-3(pk1426)	control	226	29.27 ± 0.28			9
	c36b1.5	129	19.56 ± 0.33	-33.17	<0.0001	2
rrf-3(pk1426)	control	191	17.76 ± 0.15			7
	c49h3.5	41	17.63 ± 0.18	-0.73	0.4989	2
eat-2(ad1116);rrf-3(pk1426)	control	108	30.19 ± 0.59			5
	c49h3.5	101	23.41 ± 0.36	-22.46	<0.0001	2
eat-2(ad465);rrf-3(pk1426)	control	226	29.27 ± 0.28			9
	c49h3.5	122	20.48 ± 0.21	-30.03	<0.0001	3
rrf-3(pk1426)	control	191	17.76 ± 0.15			7
	f11a10.2	97	18.39 ± 0.29	3.55	0.0063	4
eat-2(ad1116);rrf-3(pk1426)	control	108	30.19 ± 0.59			5
	f11a10.2	138	21.13 ± 0.39	-30.01	<0.0001	2
eat-2(ad465);rrf-3(pk1426)	control	226	29.27 ± 0.28			9
	f11a10.2	128	19.22 ± 0.28	-34.34	<0.0001	3
rrf-3(pk1426)	control	191	17.76 ± 0.15			7
	f32a5.7	145	15.17 ± 0.12	-14.58	<0.0001	7
eat-2(ad1116);rrf-3(pk1426)	control	108	30.19 ± 0.59			5
	f32a5.7	117	20.32 ± 0.27	-32.69	<0.0001	1
eat-2(ad465);rrf-3(pk1426)	control	226	29.27 ± 0.28			9

	f32a5.7	130	21.29 ± 0.18	-27.26	<0.0001	3
rrf-3(pk1426)	control	191	17.76 ± 0.15			7
	w04d2.6	59	17.25 ± 0.34	-2.87	0.2538	1
eat-2(ad1116);rrf-3(pk1426)	control	108	30.19 ± 0.59			5
	w04d2.6	46	19 ± 0	-37.07	<0.0001	2
eat-2(ad465);rrf-3(pk1426)	control	226	29.27 ± 0.28			9
	w04d2.6	75	20.04 ± 0.32	-31.53	<0.0001	2
rrf-3(pk1426)	control	191	17.76 ± 0.15			7
	y41e3.11	159	17.53 ± 0.18	-1.30	0.2406	9
eat-2(ad1116);rrf-3(pk1426)	control	108	30.19 ± 0.59			5
	y41e3.11	106	18.93 ± 0.53	-37.29	0.1003	5
eat-2(ad465);rrf-3(pk1426)	control	226	29.27 ± 0.28			9
	y41e3.11	124	18.44 ± 0.23	-37.00	<0.0001	3
rrf-3(pk1426)	control	191	17.76 ± 0.15			7
	f28f8.3	79	16.03 ± 0.16	-9.74	<0.0001	3
eat-2(ad1116);rrf-3(pk1426)	control	108	30.19 ± 0.59			5
	f28f8.3	102	21.57 ± 0.27	-28.55	<0.0001	5
rrf-3(pk1426)	control	205	17.8 ± 0.18			10
	f28f8.3	92	16.6 ± 0.23	-6.74	<0.0001	4
eat-2(ad465);rrf-3(pk1426)	control	153	28.92 ± 0.38			6
	f28f8.3	137	20.34 ± 0.27	-29.67	<0.0001	9
rrf-3(pk1426)	control	110	19.12 ± 0.32			3
	f56a8.6	84	15.76 ± 0.22	-17.57	<0.0001	2
eat-2(ad1116);rrf-3(pk1426)	control	93	26.23 ± 0.68			4
	f56a8.6	58	17.9 ± 0.07	-31.76	<0.0001	0
rrf-3(pk1426)	control	205	17.8 ± 0.18			10
	f56a8.6	137	18.25 ± 0.19	2.53	0.2376	1
eat-2(ad465);rrf-3(pk1426)	control	153	28.92 ± 0.38			6
	f56a8.6	77	13.52 ± 0.16	-53.25	<0.0001	2
rrf-3(pk1426)	control	191	17.76 ± 0.15			7
	k08d10.3	135	17.84 ± 0.18	0.45	0.5642	0
eat-2(ad1116);rrf-3(pk1426)	control	108	30.19 ± 0.59			5
	k08d10.3	113	22.26 ± 0.36	-26.27	<0.0001	3
rrf-3(pk1426)	control	156	17.74 ± 0.18			4
	k08d10.3	79	16.41 ± 0.28	-7.50	<0.0001	5
eat-2(ad465);rrf-3(pk1426)	control	205	30.69 ± 0.32			7

	k08d10.3	248	23.63 ± 0.2	-23.00	<0.0001	8
rrf-3(pk1426)	control	191	17.76 ± 0.15			7
	m03c11.7	73	15.86 ± 0.27	-10.70	<0.0001	3
eat-2(ad1116);rrf-3(pk1426)	control	108	30.19 ± 0.59			5
	m03c11.7	285	15.47 ± 0.14	-48.76	<0.0001	5
rrf-3(pk1426)	control	156	17.74 ± 0.18			4
	m03c11.7	104	14.44 ± 0.22	-18.60	0.0397	1
eat-2(ad465);rrf-3(pk1426)	control	205	30.69 ± 0.32			7
	m03c11.7	100	14.12 ± 0.06	-53.99	<0.0001	0
rrf-3(pk1426)	control	191	17.76 ± 0.15			7
	r07e5.14	121	18.4 ± 0.19	3.60	0.0131	2
eat-2(ad1116);rrf-3(pk1426)	control	108	30.19 ± 0.59			5
	r07e5.14	130	21.22 ± 0.3	-29.71	<0.0001	3
rrf-3(pk1426)	control	156	17.74 ± 0.18			4
	r07e5.14	135	19.68 ± 0.23	10.94	<0.0001	0
eat-2(ad465);rrf-3(pk1426)	control	205	30.69 ± 0.32			7
	r07e5.14	146	18.47 ± 0.22	-39.82	<0.0001	2
rrf-3(pk1426)	control	191	17.76 ± 0.15			7
	t04a8.6	119	15.45 ± 0.14	-13.01	<0.0001	2
eat-2(ad1116);rrf-3(pk1426)	control	108	30.19 ± 0.59			5
	t04a8.6	146	21.31 ± 0.26	-29.41	<0.0001	8
rrf-3(pk1426)	control	156	17.74 ± 0.18			4
	t04a8.6	112	16.75 ± 0.24	-5.58	<0.0001	6
eat-2(ad465);rrf-3(pk1426)	control	205	30.69 ± 0.32			7
	t04a8.6	172	21.19 ± 0.22	-30.95	<0.0001	11
rrf-3(pk1426)	control	191	17.76 ± 0.15			7
	t23f6.4	104	18.15 ± 0.26	2.20	0.0615	2
eat-2(ad1116);rrf-3(pk1426)	control	108	30.19 ± 0.59			5
	t23f6.4	54	22.26 ± 0.46	-26.27	<0.0001	3
rrf-3(pk1426)	control	191	17.76 ± 0.15			7
	t23f6.4	104	18.15 ± 0.26	2.20	0.0615	2
eat-2(ad465);rrf-3(pk1426)	control	226	29.27 ± 0.28			9
	t23f6.4	130	18.27 ± 0.19	-37.58	<0.0001	1
rrf-3(pk1426)	control	191	17.76 ± 0.15			7
	w07e6.4	84	15.61 ± 0.18	-12.11	<0.0001	0
eat-2(ad1116);rrf-3(pk1426)	control	108	30.19 ± 0.59			5

	w07e6.4	114	16.32 ± 0.23	-45.94	<0.0001	2
rrf-3(pk1426)	control	191	17.76 ± 0.15			7
	w07e6.4	84	15.61 ± 0.18	-12.11	<0.0001	0
eat-2(ad465);rrf-3(pk1426)	control	226	29.27 ± 0.28			9
	w07e6.4	151	15.93 ± 0.17	-45.58	<0.0001	6
rrf-3(pk1426)	control	205	17.8 ± 0.18			10
	y106g6d.7	168	17.36 ± 0.29	-2.47	0.8854	5
eat-2(ad1116);rrf-3(pk1426)	control	150	28.27 ± 0.4			5
	y106g6d.7	108	21.87 ± 0.27	-22.64	<0.0001	4
rrf-3(pk1426)	control	205	17.8 ± 0.18			10
	y106g6d.7	168	17.36 ± 0.29	-2.47	0.8854	5
eat-2(ad465);rrf-3(pk1426)	control	153	28.92 ± 0.38			6
	y106g6d.7	80	24.15 ± 0.53	-16.49	<0.0001	1
rrf-3(pk1426)	control	110	19.12 ± 0.32			3
	y106g6h.2	100	18.74 ± 0.22	-1.99	0.9287	2
eat-2(ad1116);rrf-3(pk1426)	control	93	26.23 ± 0.68			4
	y106g6h.2	155	22.66 ± 0.33	-13.61	<0.0001	5
rrf-3(pk1426)	control	191	17.76 ± 0.15			7
	y106g6h.2	84	23.79 ± 0.3	33.95	<0.0001	6
eat-2(ad465);rrf-3(pk1426)	control	226	29.27 ± 0.28			9
	y106g6h.2	50	25.18 ± 0.52	-13.97	<0.0001	2

Experiment 2

**Genotype**	**RNAi**	**n**	**Mean LS ± SEM (Days)**	**% Change w.r.t. Control RNAi**	***P*- value (control Vs RNAi)**	**Censored population**
rrf-3(pk1426)	control	124	19.85 ± 0.29			2
	b0035.12	195	18.02 ± 0.2	-9.22	<0.0001	6
eat-2(ad1116);rrf-3(pk1426)	control	234	26.64 ± 0.32			8
	b0035.12	152	18.82 ± 0.21	-29.35	<0.0001	2
eat-2(ad465);rrf-3(pk1426)	control	214	28.46 ± 0.39			5
	b0035.12	172	20.43 ± 0.21	-28.22	<0.0001	3
rrf-3(pk1426)	control	124	19.85 ± 0.29			2
	c36b1.5	105	15 ± 0.16	-24.43	<0.0001	3
eat-2(ad1116);rrf-3(pk1426)	control	234	26.64 ± 0.32			8
	c36b1.5	219	15.82 ± 0.13	-40.62	<0.0001	2

eat-2(ad465);rrf-3(pk1426)	control	214	28.46 ± 0.39			5
	c36b1.5	203	15.55 ± 0.22	-45.36	<0.0001	5
rrf-3(pk1426)	control	124	19.85 ± 0.29			2
	c49h3.5	169	16.88 ± 0.17	-14.96	<0.0001	2
eat-2(ad1116);rrf-3(pk1426)	control	234	26.64 ± 0.32			8
	c49h3.5	205	24.12 ± 0.27	-9.46	<0.0001	12
eat-2(ad465);rrf-3(pk1426)	control	214	28.46 ± 0.39			5
	c49h3.5	107	19.07 ± 0.24	-32.99	<0.0001	3
rrf-3(pk1426)	control	124	19.85 ± 0.29			2
	f11a10.2	115	16.92 ± 0.23	-14.76	<0.0001	2
eat-2(ad1116);rrf-3(pk1426)	control	234	26.64 ± 0.32			8
	f11a10.2	179	17.51 ± 0.14	-34.27	<0.0001	1
eat-2(ad465);rrf-3(pk1426)	control	214	28.46 ± 0.39			5
	f11a10.2	109	20.1 ± 0.16	-29.37	<0.0001	2
rrf-3(pk1426)	control	124	19.85 ± 0.29			2
	f32a5.7	230	17.39 ± 0.13	-12.39	<0.0001	5
eat-2(ad1116);rrf-3(pk1426)	control	234	26.64 ± 0.32			8
	f32a5.7	156	18.02 ± 0.11	-32.36	<0.0001	1
eat-2(ad465);rrf-3(pk1426)	control	214	28.46 ± 0.39			5
	f32a5.7	164	20.14 ± 0.19	-29.23	<0.0001	2
rrf-3(pk1426)	control	124	19.85 ± 0.29			2
	w04d2.6	230	17.16 ± 0.14	-13.55	<0.0001	2
eat-2(ad1116);rrf-3(pk1426)	control	234	26.64 ± 0.32			8
	w04d2.6	136	18.22 ± 0.12	-31.61	<0.0001	0
eat-2(ad465);rrf-3(pk1426)	control	214	28.46 ± 0.39			5
	w04d2.6	170	20.46 ± 0.29	-28.11	<0.0001	3
rrf-3(pk1426)	control	124	19.85 ± 0.29			2
	y41e3.11	153	18.42 ± 0.16	-7.20	<0.0001	2
eat-2(ad1116);rrf-3(pk1426)	control	234	26.64 ± 0.32			8
	y41e3.11	158	18.24 ± 0.1	-31.53	<0.0001	0
eat-2(ad465);rrf-3(pk1426)	control	214	28.46 ± 0.39			5
	y41e3.11	202	18.5 ± 0.19	-35.00	<0.0001	1
rrf-3(pk1426)	control	191	17.76 ± 0.15			7
	f28f8.3	133	18.24 ± 0.14	-8.11	<0.0001	5
eat-2(ad1116);rrf-3(pk1426)	control	108	30.19 ± 0.59			5
	f28f8.3	106	20.94 ± 0.22	-21.40	<0.0001	2

rrf-3(pk1426)	control	156	17.74 ± 0.18			4
	f28f8.3	165	17.84 ± 0.21	0.56	<0.0001	1
eat-2(ad465);rrf-3(pk1426)	control	205	30.69 ± 0.32			7
	f28f8.3	240	20.3 ± 0.26	-33.85	<0.0001	3
rrf-3(pk1426)	control	124	19.85 ± 0.29			2
	f56a8.6	134	18.19 ± 0.19	-8.36	<0.0001	2
eat-2(ad1116);rrf-3(pk1426)	control	234	26.64 ± 0.32			8
	f56a8.6	133	23.9 ± 0.29	-10.29	<0.0001	1
rrf-3(pk1426)	control	124	19.85 ± 0.29			2
	f56a8.6	134	18.19 ± 0.19	-8.36	<0.0001	2
eat-2(ad465);rrf-3(pk1426)	control	214	28.46 ± 0.39			5
	f56a8.6	86	20.66 ± 0.23	-27.41	<0.0001	3
rrf-3(pk1426)	control	124	19.85 ± 0.29			2
	k08d10.3	102	17.6 ± 0.23	-11.34	<0.0001	3
eat-2(ad1116);rrf-3(pk1426)	control	234	26.64 ± 0.32			8
	k08d10.3	195	17.67 ± 0.16	-33.67	<0.0001	5
rrf-3(pk1426)	control	124	19.85 ± 0.29			2
	k08d10.3	102	17.6 ± 0.23	-11.34	<0.0001	3
eat-2(ad465);rrf-3(pk1426)	control	214	28.46 ± 0.39			5
	k08d10.3	135	22.04 ± 0.29	-22.56	<0.0001	1
rrf-3(pk1426)	control	124	19.85 ± 0.29			2
	m03c11.7	66	14.59 ± 0.15	-26.50	<0.0001	0
eat-2(ad1116);rrf-3(pk1426)	control	234	26.64 ± 0.32			8
	m03c11.7	120	15.13 ± 0.18	-43.21	<0.0001	2
rrf-3(pk1426)	control	124	19.85 ± 0.29			2
	m03c11.7	66	14.59 ± 0.15	-26.50	<0.0001	0
eat-2(ad465);rrf-3(pk1426)	control	214	28.46 ± 0.39			5
	m03c11.7	262	13.34 ± 0.11	-53.13	<0.0001	3
rrf-3(pk1426)	control	124	19.85 ± 0.29			2
	r07e5.14	181	22.36 ± 0.27	12.64	<0.0001	3
eat-2(ad1116);rrf-3(pk1426)	control	234	26.64 ± 0.32			8
	r07e5.14	248	20.47 ± 0.24	-23.16	<0.0001	2
rrf-3(pk1426)	control	124	19.85 ± 0.29			2
	r07e5.14	181	22.36 ± 0.27	12.64	<0.0001	3
eat-2(ad465);rrf-3(pk1426)	control	214	28.46 ± 0.39			5
	r07e5.14	150	21.53 ± 0.24	-24.35	<0.0001	5

rrf-3(pk1426)	control	124	19.85 ± 0.29			2
	t04a8.6	206	18.43 ± 0.17	-7.15	<0.0001	6
eat-2(ad1116);rrf-3(pk1426)	control	234	26.64 ± 0.32			8
	t04a8.6	220	19.03 ± 0.17	-28.57	<0.0001	1
rrf-3(pk1426)	control	124	19.85 ± 0.29			2
	t04a8.6	206	18.43 ± 0.17	-7.15	<0.0001	6
eat-2(ad465);rrf-3(pk1426)	control	214	28.46 ± 0.39			5
	t04a8.6	188	19.3 ± 0.26	-32.19	<0.0001	8
rrf-3(pk1426)	control	110	19.12 ± 0.32			3
	t23f6.4	83	16.98 ± 0.32	-11.19	<0.0001	7
eat-2(ad1116);rrf-3(pk1426)	control	93	26.23 ± 0.68			4
	t23f6.4	123	17.99 ± 0.24	-31.41	<0.0001	6
rrf-3(pk1426)	control	124	19.85 ± 0.29			2
	t23f6.4	79	18.68 ± 0.26	-5.89	0.0026	4
eat-2(ad465);rrf-3(pk1426)	control	214	28.46 ± 0.39			5
	t23f6.4	193	16.85 ± 0.26	-40.79	<0.0001	2
rrf-3(pk1426)	control	110	19.12 ± 0.32			3
	w07e6.4	90	17.13 ± 0.19	-10.41	0.0001	4
eat-2(ad1116);rrf-3(pk1426)	control	93	26.23 ± 0.68			4
	w07e6.4	100	19.17 ± 0.32	-26.92	<0.0001	1
rrf-3(pk1426)	control	110	19.12 ± 0.32			3
	w07e6.4	90	17.13 ± 0.19	-10.41	0.0001	4
eat-2(ad465);rrf-3(pk1426)	control	89	28.08 ± 0.75	47.63	<0.0001	1
	w07e6.4	79	20.13 ± 0.44	-28.31	<0.0001	2
rrf-3(pk1426)	control	124	19.85 ± 0.29			2
	y106g6d.7	164	18.17 ± 0.14	-8.46	<0.0001	10
eat-2(ad1116);rrf-3(pk1426)	control	234	26.64 ± 0.32			8
	y106g6d.7	177	24.21 ± 0.31	-9.12	<0.0001	12
rrf-3(pk1426)	control	191	17.76 ± 0.15			7
	y106g6d.7	96	18.97 ± 0.27	6.81	<0.0001	8
eat-2(ad465);rrf-3(pk1426)	control	226	29.27 ± 0.28			9
	y106g6d.7	38	24.32 ± 0.7	-16.91	<0.0001	0
rrf-3(pk1426)	control	101	19.02 ± 0.28			8
	y106g6h.2	86	17.31 ± 0.31	-8.99	0.0001	2
eat-2(ad1116);rrf-3(pk1426)	control	176	34.27 ± 0.52			6
	y106g6h.2	61	24.23 ± 0.48	-29.30	<0.0001	1

rrf-3(pk1426)	control	110	19.12 ± 0.32			3
	y106g6h.2	100	18.74 ± 0.22	-1.99	0.9287	4
eat-2(ad465);rrf-3(pk1426)	control	89	28.08 ± 0.75			1
	y106g6h.2	93	24.26 ± 0.48	-13.60	<0.0001	3

Supplementary Table 3: Details of bDR life span analysis of *hrpu-1* RNAi as shown in Figure 3D

BDR life span

Genotype	RNAi	n	Mean LS ± SEM (Days)	% Change w.r.t. O.D. 3.0	*P*-value w.r.t. O.D. 3.0	Censored population
**WT**						
	**Control (O.D. 3.0)**	45	26.31 ± 0.52			0
	**Control (O.D. 1.0)**	52	37.38 ± 0.90	42.08	<0.0001	0
	**Control (O.D. 0.5)**	52	43.98 ± 0.93	67.16	<0.0001	0
	**Control (O.D. 0.25)**	51	42.04 ± 0.79	59.79	<0.0001	0
	**Control (O.D. 0.125)**	52	36.54 ± 0.77	38.88	<0.0001	1
**WT**						
	***hrpu-1* (O.D. 3.0)**	51	22.84 ± 0.65			0
	***hrpu-1* (O.D. 1.0)**	55	24.22 ± 0.54	6.04	0.1738	0
	***hrpu-1* (O.D. 0.5)**	50	26.04 ± 0.66	14.01	0.0006	0
	***hrpu-1* (O.D. 0.25)**	59	26.49 ± 0.64	15.98	0.0002	0
	***hrpu-1* (O.D. 0.125)**	55	30.69 ± 0.89	34.37	<0.0001	1
**WT**						
	**Control (O.D. 3.0)**	37	30.00 ± 0.79			0
	**Control (O.D. 1.0)**	35	38.00 ± 1.21	26.67	<0.0001	0
	**Control (O.D. 0.5)**	34	41.53 ± 1.32	38.43	<0.0001	0
	**Control (O.D. 0.25)**	36	36.06 ± 1.55	20.20	<0.0001	0
	**Control (O.D. 0.125)**	35	36.31 ± 1.18	21.03	<0.0001	0
**WT**						
	***hrpu-1* (O.D. 3.0)**	32	25.59 ± 0.74			0
	***hrpu-1* (O.D. 1.0)**	24	27.92 ± 0.70	9.11	0.0446	0
	***hrpu-1* (O.D. 0.5)**	32	33.56 ± 0.71	31.14	<0.0001	0
	***hrpu-1* (O.D. 0.25)**	27	26.63 ± 1.25	4.06	0.1746	0
	***hrpu-1* (O.D. 0.125)**	23	26.48 ± 1.92	3.48	0.0338	0

Supplementary Table 4: Life span analysis in *smg-2* deficient conditions
BDR life span in *smg-2* mutant

Genotype	RNAi	n	Mean LS ± SEM (Days)	% Change w.r.t. O.D. 3.0	*P*-value w.r.t. O.D. 3.0	Censored population
him-5(e1490)						
	**OP50 (O.D. 3.0)**	42	25.19 ± 0.76			0
	**OP50 (O.D. 1.0)**	26	25.08 ± 1.49	-0.44	0.5311	0
	**OP50 (O.D. 0.5)**	45	31.96 ± 0.99	26.88	<0.0001	0
	**OP50 (O.D. 0.25)**	43	23.53 ± 1.06	-6.59	0.6185	0
	**OP50 (O.D. 0.125)**	42	24.86 ± 0.96	-1.31	0.787	0
	**OP50 (O.D. 0.016)**	43	18.84 ± 0.92	-25.21	<0.0001	0
smg-2(e2008); him-5(e1490)						
	**OP50 (O.D. 3.0)**	42	23.48 ± 0.89			0
	**OP50 (O.D. 1.0)**	43	25.67 ± 0.88	9.33	0.1235	0
	**OP50 (O.D. 0.5)**	46	25.48 ± 0.85	8.52	0.1467	0
	**OP50 (O.D. 0.25)**	46	25.83 ± 0.99	10.01	0.063	0
	**OP50 (O.D. 0.125)**	33	22.79 ± 1.15	-2.94	0.7923	0
	**OP50 (O.D. 0.016)**	44	23.32 ± 0.77	-0.68	0.6501	1
him-5(e1490)						
	**OP50 (O.D. 3.0)**	46	29.17 ± 0.64			0
	**OP50 (O.D. 1.0)**	46	35.15 ± 0.99	20.50	<0.0001	0
	**OP50 (O.D. 0.5)**	48	37.42 ± 0.99	28.28	<0.0001	0
	**OP50 (O.D. 0.25)**	43	29.98 ± 1.16	2.78	0.129	0
	**OP50 (O.D. 0.125)**	44	25.89 ± 1.21	-11.24	0.2088	0
	**OP50 (O.D. 0.016)**	40	23.58 ± 1.19	-19.16	0.004	0
smg-2(e2008); him-5(e1490)						
	**OP50 (O.D. 3.0)**	47	26.30 ± 0.65			0
	**OP50 (O.D. 1.0)**	47	27.62 ± 0.66	5.02	0.0989	0
	**OP50 (O.D. 0.5)**	44	29.07 ± 0.58	10.53	0.0017	0
	**OP50 (O.D. 0.25)**	44	27.20 ± 0.74	3.42	0.1861	0
	**OP50 (O.D. 0.125)**	44	27.14 ± 0.73	3.19	0.2368	0
	**OP50 (O.D. 0.016)**	43	27.02 ± 0.82	2.74	0.2542	1

him-5(e1490)						
	**OP50 (O.D. 3.0)**	44	22.48 ± 0.98			0
	**OP50 (O.D. 1.0)**	44	26.70 ± 1.28	18.77	0.0006	0
	**OP50 (O.D. 0.5)**	49	30.29 ± 1.43	34.74	<0.0001	0
	**OP50 (O.D. 0.25)**	48	27.63 ± 1.58	22.91	<0.0001	0
	**OP50 (O.D. 0.125)**	43	28.65 ± 1.20	27.45	<0.0001	0
	**OP50 (O.D. 0.016)**	45	23.69 ± 1.22	5.38	0.1159	2
smg-2(e2008); him-5(e1490)						
	**OP50 (O.D. 3.0)**	43	21.30 ± 0.65			0
	**OP50 (O.D. 1.0)**	46	22.17 ± 0.56	4.08	0.4107	0
	**OP50 (O.D. 0.5)**	47	23.21 ± 0.84	8.97	0.012	0
	**OP50 (O.D. 0.25)**	45	20.98 ± 0.69	-1.50	0.8274	0
	**OP50 (O.D. 0.125)**	44	22.16 ± 0.79	4.04	0.179	0
	**OP50 (O.D. 0.016)**	36	20.08 ± 0.84	-5.73	0.4149	0

him-5(e1490)						
	**OP50 (O.D. 3.0)**	47	24.40 ± 0.60			0
	**OP50 (O.D. 1.0)**	45	31.69 ± 0.82	29.88	<0.0001	0
	**OP50 (O.D. 0.5)**	45	38.96 ± 0.93	59.67	<0.0001	0
	**OP50 (O.D. 0.25)**	45	36.04 ± 1.01	47.70	<0.0001	0
	**OP50 (O.D. 0.125)**	33	35.88 ± 1.31	47.05	<0.0001	0
	**OP50 (O.D. 0.016)**	40	34.35 ± 1.34	40.78	<0.0001	0
smg-2(e2008); him-5(e1490)						
	**OP50 (O.D. 3.0)**	43	22.00 ± 0.73			0
	**OP50 (O.D. 1.0)**	46	23.07 ± 0.61	4.86	0.4107	0
	**OP50 (O.D. 0.5)**	47	24.02 ± 0.95	9.18	0.012	0
	**OP50 (O.D. 0.25)**	45	21.67 ± 0.80	-1.50	0.8274	0
	**OP50 (O.D. 0.125)**	45	23.29 ± 0.92	5.86	0.1201	0
	**OP50 (O.D. 0.016)**	37	21.08 ± 1.03	-4.18	0.6546	1

***smg-2* RNAi life span**

Genotype	RNAi	n	Mean LS ± SEM (Days)	% Change w.r.t. Control RNAi	*P-* value (control Vs RNAi)	Censored population
**WT**						
	control	66	18.83 ± 0.39			2
	smg-2	68	16.88 ± 0.36	-10.36	<0.0001	1
eat-2(ad1116)						
	control	86	27.52 ± 0.39			1
	smg-2	85	20.15 ± 0.18	-26.78	<0.0001	4
**WT**						
	control	70	19.60 ± 0.44			0
	smg-2	96	17.08 ± 0.11	-12.86	<0.0001	1
eat-2(ad1116)						
	control	91	23.93 ± 0.23			3
	smg-2	93	18.04 ± 0.2	-24.61	<0.0001	2
**WT**						
	control	70	21.5 ± 0.36			1
	smg-2	136	19.66 ± 0.24	-8.56	0.0001	2
eat-2(ad1116)						
	control	58	26.78 ± 0.68			0
	smg-2	37	19.62 ± 0.54	-26.06	<.0001	0
